# Effects of robotic-assisted upper extremity therapy for stroke patients in different recovery phases: a systematic review and meta-analysis

**DOI:** 10.3389/fneur.2025.1640522

**Published:** 2026-01-12

**Authors:** Lulu Wang, Xiaoling Li, Yan Liu, Enyu Zhang, Cuiting Li

**Affiliations:** Department of Rehabilitation Medicine, Lanzhou University Second Hospital, Lanzhou, Gansu, China

**Keywords:** stroke, upper extremity, robotics, rehabilitation, neuronal plasticity, randomized controlled trial, meta-analysis

## Abstract

**Objective:**

To systematically evaluate the efficacy of robotic-assisted therapy on upper extremity recovery after stroke through stratified meta-analysis, assessing the influence of disease phase, injury severity, device type, training parameters, and follow-up duration.

**Methods:**

We conducted a systematic literature search of PubMed, Embase, Cochrane Library, and Web of Science from inception to July 2025 for randomized controlled trials (RCTs) comparing robotic-assisted with conventional upper limb therapy in adult stroke patients.

**Results:**

Analysis of 42 RCTs (*n* = 1,678) demonstrated that robotic-assisted therapy significantly improved motor function (Fugl-Meyer Upper Extremity Motor Assessment (FMA-UE)), grip strength (GS), activities of daily living (Modified Barthel Index (MBI)), and social participation (Stroke Impact Scale (SIS)) compared to conventional therapy. Crucially, patients in the subacute phase and those with severe impairment achieved clinically meaningful recovery, with improvements in motor function (FMA-UE: WMD = 8.82, 95% CI (4.42, 13.23)) and activities of daily living (MBI: WMD = 8.00, 95% CI (4.96, 11.03)) exceeding established Minimal Clinically Important Difference (MCID) thresholds.

**Conclusion:**

Robotic-assisted therapy significantly improves upper extremity motor function and activities of daily living after stroke, with clinically meaningful gains particularly evident in subacute patients and those with severe impairment. Benefits in grip strength and social participation were statistically significant but of uncertain clinical importance due to methodological limitations. These findings support a stratified rehabilitation approach, prioritizing subacute and severely impaired patients for robotic intervention to maximize functional outcomes.

**Systematic review registration:**

https://www.crd.york.ac.uk/PROSPERO/view/CRD42025640276, CRD42025640276.

## 1 Introduction

Globally, stroke ranks as the second leading cause of mortality and third leading contributor to disability-adjusted life years, imposing substantial socioeconomic burdens—particularly in low- and middle-income countries (1). Over 80% of acute stroke survivors exhibit persistent upper limb dysfunction (2), with residual deficits continuing to impair quality of life up to 4 years post-stroke (3), underscoring the urgent need for effective rehabilitation strategies.

Upper extremity rehabilitation robotics, first conceptualized in 1978 (4), have evolved into clinically validated tools for neurorehabilitation. Their capacity to deliver high-intensity, repeatable protocols with precise kinematic control and safety profiles has driven widespread adoption in stroke rehabilitation (5, 6). While meta-analyses demonstrate non-inferiority to dose-matched conventional therapies (5, 7, 8), critical knowledge gaps persist. Existing studies predominantly focus on isolated recovery phases, neglecting systematic evaluation across the entire rehabilitation continuum. Furthermore, the neurophysiological mechanisms underpinning recovery during robotic intervention are an area of active investigation. Recent evidence suggests that robotic training can promote cortical reorganization and strengthen corticospinal tract integrity, with neuroimaging and electrophysiological studies beginning to delineate these plasticity mechanisms (2, 9).

This study therefore sets out to address these gaps through a comprehensive meta-analysis that systematically evaluates the effects of robotic-assisted therapy across different disease stages, injury severities, robotic types, and training parameters. We further synthesize evidence on its relationship with neurophysiological mechanisms to explore potential bases for functional recovery. The findings aim to guide a more stratified application of robotic rehabilitation and highlight evidence gaps for future research.

## 2 Materials and methods

This systematic review was conducted in accordance with the Preferred Reporting Items for Systematic Reviews and Meta-Analyses (PRISMA 2020) guidelines (see Supplementary Table S1) and prospectively registered in the PROSPERO international prospective register of systematic reviews (Registration ID: CRD42025640276).

### 2.1 Search strategy and selection criteria

Two independent investigators (WLL and ZEY) systematically searched PubMed, Embase, Cochrane Library, and Web of Science electronic databases from database inception through September 2, 2024, with a further updated search on July 21, 2025. The search strategy incorporated controlled vocabulary (MeSH/Emtree terms) and free-text keywords addressing stroke rehabilitation, upper extremity, robotics and randomized controlled trial (RCT). Full search syntaxes for all databases are documented in Supplementary Table S2.

### 2.2 Inclusion and exclusion criteria

#### 2.2.1 Inclusion criteria

The systematic review employed the PICO (Population, Intervention, Control, Outcomes) framework to establish selection criteria: (1) Population: Adults (≥18 years) with radiologically confirmed unilateral hemispheric stroke and upper limb motor and function impairment; (2) Intervention: Experimental interventions involving robot-assisted upper limb rehabilitation utilizing electromechanical devices, including but not limited to end-effector robotics (EE), exoskeletal robot devices (EXO), or soft robotic gloves (SRG); (3) Control: At least one control group receiving conventional rehabilitation therapy (e.g., physical therapy, occupational therapy, task-oriented therapy, intensive rehabilitation therapy); (4) Outcomes: outcome measures included the Fugl-Meyer Upper Extremity Motor Assessment (FMA-UE), Modified Ashworth Scale (MAS), grip strength (GS), Modified Barthel Index (MBI), and Stroke Impact Scale (SIS), Fugl-Meyer Upper Extremity Motor Assessment -proximal/ shoulder and elbow coordination (FMA-SE), Fugl-Meyer Upper Extremity Motor Assessment -distal/ wrist and hand function (FMA-WH), Fugl-Meyer Upper Extremity Motor Assessment -hand dexterity (FMA-H), Motor Activity Log - amount of use (MAL-AOU), and Motor Activity Log - quality of movement (MAL-QOM); (5) Randomized controlled trials (RCTs).

#### 2.2.2 Exclusion criteria

Studies were excluded if they met any of the following criteria: (1) Interventions involving group-, family-, or self-guided therapy; (2) Bilateral upper limb treatment in either experimental or control groups; (3) Insufficient outcome data for effect size calculation; (4) Robotic-assisted therapy combined with adjunct interventions (e.g., repetitive transcranial magnetic stimulation (rTMS), virtual reality (VR), or brain-computer interfaces (BCI)); (5) Comparative studies using distinct robotic device types across intervention arms; (6) Mixed stroke stage cohorts (acute/subacute/chronic) without stratified analysis; (7) Crossover study designs.

#### 2.2.3 Operational definitions

Stroke phases were classified as: (1) Acute: ≤1 month post-onset; (2) Subacute: 1–6 months; (3) Chronic: ≥6 months (10, 11).

Note: This staging system may differ from source studies due to temporal reclassification.

#### 2.2.4 Analytical considerations

Sample size and attrition rates were calculated exclusively from participants completing final outcome assessments.

### 2.3 Outcome measures

Outcome measures were aligned with the International Classification of Functioning, Disability and Health (ICF) framework (12), encompassing: (1) Body Functions & Structures: Primary outcome measures included FMA-UE to evaluate motor recovery, MAS for spasticity assessment, and grip strength quantified via dynamometry. Secondary measures comprised domain-specific FMA-SE, FMA-WH, and FMA-H; (2) Activities & Participation: Primary functional outcomes included MBI to assess activities of daily living and SIS for multidimensional stroke-related disability evaluation. Secondary measures focused on real-world upper limb performance, utilizing MAL-AOU to quantify functional engagement frequency and MAL-QOM to characterize movement efficacy during daily tasks.

### 2.4 Data extraction and quality assessment

#### 2.4.1 Data extraction

Two investigators (WLL and ZEY) conducted dual independent screening of titles/abstracts against predefined eligibility criteria, followed by full-text assessments. Discrepancies were resolved through consensus discussions with a senior researcher (LXL).

A standardized extraction template captured: (1) Study descriptors: Title, first author, publication year, location; (2) Participant characteristics: Age, sex, post-stroke duration (days), baseline motor scores; (3) Intervention parameters: Robotic device type, session length (minutes), total duration length, follow-up period; (4) Outcome metrics: Pre/post-intervention scores with variance measures for all specified scales.

Missing outcome data were addressed by contacting corresponding authors via email. Studies with incomplete datasets unresponsive to data requests were systematically excluded from quantitative synthesis.

#### 2.4.2 Quality assessment

Two independent reviewers (LY and LCT) evaluated methodological quality using the Cochrane Risk of Bias Tool for Randomized Trials (RoB 2.0), assessing five domains: randomization process, deviations from intended interventions, missing outcome data, outcome measurement, and selective reporting. Inter-rater discrepancies were resolved through consensus adjudication by a third reviewer (LXL).

To assess the robustness of our findings, a pre-specified sensitivity analysis was performed by excluding studies judged to be at high overall risk of bias. The impact of this exclusion on both baseline characteristics and pooled effect estimates was rigorously evaluated.

### 2.5 Data synthesis and statistical analysis

All analyses were performed using Stata 18 (StataCorp LLC, College Station, TX, USA). Continuous variables are presented as means with standard deviations (SD). Effect sizes were calculated as weighted mean differences (WMD) with 95% confidence intervals (95% CI) for outcomes sharing identical measurement scales. For outcomes with heterogeneous metrics, standardized mean differences (SMD) with 95% CI were computed.

Heterogeneity was quantified using the *I*^2^ statistic and Cochran’s Q test. A fixed-effects model was applied when *I*^2^ ≤ 50% with Q test *p* ≥ 0.05; otherwise, a random-effects model was implemented. In cases of discordance between metrics (*I*^2^ vs. Q test), *I*^2^ thresholds governed model selection.

Publication bias was evaluated through Egger’s linear regression test and funnel plot asymmetry analysis. Significant bias (*p* < 0.05) triggered supplementary analyses: (1) Subgroup stratification; (2) Trim-and-fill imputation for missing studies; (3) Meta-regression examining moderator variables; (4) Leave-one-out sensitivity testing.

### 2.6 Reclassification protocol for stroke phases criteria

To address inconsistent definitions of stroke phases across studies, we implemented a standardized reclassification protocol based solely on the time from stroke onset to intervention.

Temporal data were extracted and harmonized using a predefined hierarchy: reported means or medians were prioritized; for bounded ranges, midpoints were calculated; for unbounded ranges, conservative boundary values were applied. All values were standardized to days (conversion factor: 1 month ≈ 30.44 days) (13).

We adopted a commonly used clinical framework for phase definitions (acute: ≤1 month; subacute: 1–6 months; chronic: ≥6 months) (10, 11). To empirically calibrate and validate these thresholds against our dataset, the distribution of the harmonized onset times across all participants was characterized using descriptive statistics (median and IQR). This analysis confirmed that the predefined thresholds effectively captured distinct phases within the cohort’s empirical distribution, as detailed in the Results section. All computational procedures were implemented programmatically in Python (v3.9; Python Software Foundation) to ensure reproducibility.

### 2.7 Sensitivity analysis for baseline heterogeneity

Upon detection of statistically significant heterogeneity in baseline characteristics (*p* < 0.05), meta-regression analysis was performed to quantify covariate-outcome associations through regression coefficients (*β*) with 95% CI and to compute covariate-adjusted pooled effect estimates (Hedge’s g with 95% CI). The robustness of statistically significant associations (*β* ≠ 0, *p* < 0.05) was subsequently assessed using leave-one-out sensitivity analysis.

## 3 Results

### 3.1 Study selection and characteristics

**Figure 1:**
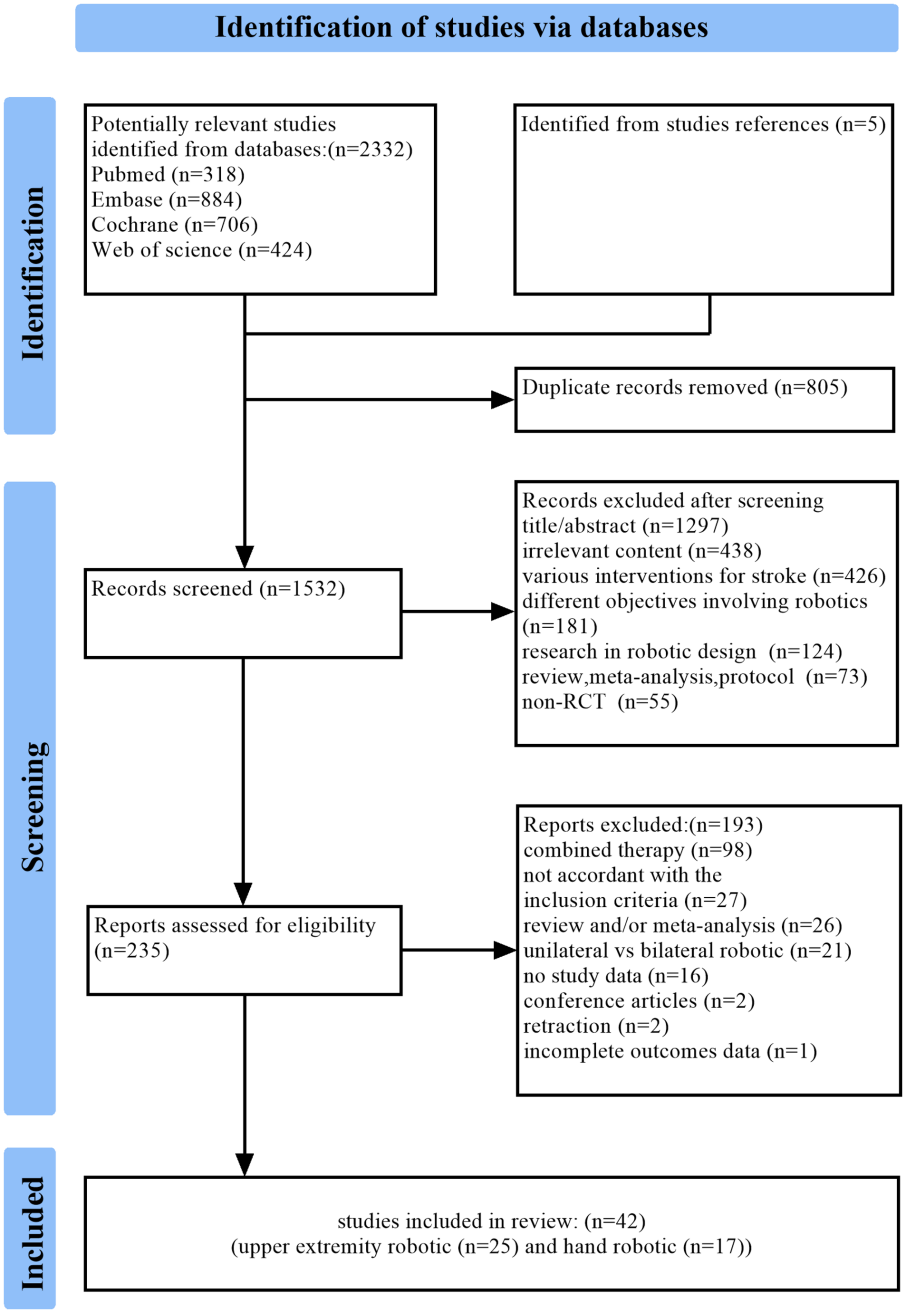
The detailed flowchart of the selection procedure. The “Irrelevant content” designation in this figure applies to studies involving: (a) robotic brain-computer interfaces, (b) cardiopulmonary rehabilitation, (c) gait analysis, (d) assistive technologies, and (e) surgical robotics, among other unrelated research domains.

The systematic search identified 2,332 records from databases, supplemented by 5 additional studies from reference lists, yielding 2,337 total entries. After removing 805 duplicates, 1,532 studies underwent title/abstract screening, with 1,297 excluded for irrelevance. Full-text review of 235 potentially eligible articles resulted in 193 exclusions (reasons detailed in Figure 1), culminating in 42 studies for final inclusion:25 investigating upper extremity robot-assisted rehabilitation and 17 evaluating hand-specific robotic devices.

The PRISMA-compliant selection flowchart (Figure 1) documents attrition at each phase.

The meta-analysis included 1,678 stroke patients from 42 studies, comprising 807 participants in robotic-assisted rehabilitation groups (mean age 60.64 ± 12.52 years) and 776 in conventional therapy groups (mean age 60.59 ± 12.68 years). Sex distribution was reported for 1,646 patients (98.10% completeness): (1) Robotic-assisted groups: 531 males (68.25%), 247 females (31.75%); (2) Conventional groups: 485 males (64.67%), 265 females (35.33%).

Four three-arm trials (A4 (14) (*n* = 13), A14 (15) (*n* = 16), A17 (16) (*n* = 16), A19 (17) (*n* = 50); total *n* = 95) were included, with only intervention-comparator pairs meeting PICO criteria retained for analysis. Complete demographic data are tabulated in Table 1.

**Table 1:** Characteristics of included studies.

Study		Location	Age (EG/CG)	Gender (EG(M/F)/CG(M/F))	Robotic and type	*n* (EG/CG)	Days since stroke onset and phase (EG/CG/phase)	Characteristics interventions		Outcomes
								Session and duration length	Follow-up¿	
A1 (29)	Tekin et al.	Turkey	67.93 ± 2.93/68.47 ± 3.33	(8/7)/(7/8)	The ReoGo system/EE	15//15	both≥180 days/chronic	60 min RT + 45 min CT/session/d, 5d/w for 4 weeks, total 20 sessions		FMA-UE
A2 (18)	Liu et al.	China	65.8 ± 9.0/66.1 ± 6.9	(20/4)/(15/9)	ArmMotus M2/EE	24/24	118.72 ± 76.1/106.54 ± 82.19/subacute	30 min/session/d, 5d/w for 4 weeks, total 20 sessions		FMA-UE, FMA-SE, FMA-H, MBI
A3 (19)	Bhattacharjee et al.	India	53.32 ± 9.93/53.23 ± 10.51	(13/9)/ (12/10)	Armeo Spring/EXO	22/22	126.02 ± 36.83/124.50 ± 47.79/subacute	30 minRT+30 CT min/session/d, 5 days/w for 3 weeks, total 15 sessions	1.5, 3	FMA-UE, MAS, SIS
A4 (14)	Feingold-Polak et al.	Israel	54.3 ± 12.7/57.3 ± 12.7	(6/5)/ (6/3)	Pepper robot	11/9	108 ± 56/ 92 ± 42/ subacute	45 ~ 60 min/session, 2 ~ 3 sessions/w for 5 ~ 7 weeks, total 15 sessions		FMA-UE, SIS, MAL-AOU, MAL-QOM
A5 (30)	Chen et al.	China	50.1 ± 10.0/53.7 ± 9.4	(34/6)/ (33/7)	Armule/EXO	40/40	50.3 ± 40.3/58.9 ± 35.4/subacute	45 min/session/d, 5 days/w for 4 weeks, total 20 sessions		FMA-UE, FMA-SE, FMA-WH, MBI
A6 (20)	Lin et al.	China	59.37 ± 10.96/58.72 ± 12.89	(60/22)/ (64/22)	FLEXO-Arm1 robot/EXO	82/86	142.30 ± 162.84/158.23 ± 178.20/chronic	EG = 30 min/session/d; CG = 30 min PT + 30 min OT/session/d, both 5 days/w for 3 weeks, total 15 sessions		FMA-UE, FMA-SE, MBI
A7 (21)	Chen et al.	China	47.10 ± 11.11/54.90 ± 14.49	(10/0)/ (7/3)	robotic exoskeleton assisted EAMT therapy/EXO	10/10	74.90 ± 54.52/50.10 ± 38.24/subacute	45 min/session/d, 5 days/w for 4 weeks, total 20 sessions		FMA-UE, FMA-SE, FMA-WH, MBI
A8 (31)	Xu et al.	China	62.2 ± 10.1/60.7 ± 10.6	(15/5)/ (14/6)	Fourier M2/EE	20/20	51.0 ± 19.1/47.2 ± 24.0/subacute	EG = 20 min RT + 20 min CT/session/d; CG = 40 min CT/session/d, both 5 days/w for 6 weeks, total 30 sessions		FMA-UE, MBI
A9 (32)	Franceschini et al.	Italy	70.0 ± 8.7/74.0 ± 8.9	(12/13)/ (14/9)	InMotion2/EE	25/23	30.64 ± 3.92/31 ± 3.17/subacute	45 min/session/d, 5 days/w for 6 weeks, total 30 sessions	6	FMA-UE
A10 (42)	Daunraviciene et al.	Lithuania	65.88 ± 4.87/65.47 ± 4.05	(11/6)/ (11/6)	Armeo Spring/EXO	17/17	60.48 ± 24.71/67.55 ± 47.46/subacute	EG = 30 min RT + 30 min CT/session/d; CG = 36 ~ 60 min CT + 30 min CT/session/d, both 5 days/w, total 10 sessions		FMA-UE, MAS
A11 (33)	Lee et al.	Korea	52.07 ± 14.07/50.27 ± 11.17	(8/7)/ (11/4)	REJOYCE robot/EE	15/15	chronic	EG = 30 min RT + 30 min CT/session/d; CG = 30 min CT + 30 min CT/session/d, both 5 days/w for 8 weeks, total 40 sessions		FMA-UE, MBI
A12 (43)	Lee et al.	Korea	55.76 ± 13.60/57.88 ± 11.12	(14/11)/ (12/13)	Neuro - X /EE	25/25	15.40 ± 8.05/14.40 ± 6.95/acute	EG = 30 min RT/session+30 min CT/session,1 session/d; CG = 30 min CT/session, 2 sessions/d, both 5 days/w for 2 weeks, total 20 sessions		GS
A13 (22)	Dimkic Tomic et al.	Serbia	56.5 ± 7.4/58.3 ± 5.2	(12/1)/(9/4)	ArmAssist/EE	13/13	< 3 months/subacute	EG = 30minRT + 30CT min/session/d; CG = 30minCT + 30CT min/session/d, both 5 days/w for 3 weeks, total 15 sessions		FMA-UE, FMA-SE
A14 (15)	Barker et al.	Australia	52.4 ± 15.6/51.2 ± 15.0	(12/5)/ (11/6)	SMART Arm/EE	17/17	43.9 ± 21.7/34.7 ± 31.2/subacute	60 min/session/d, 5 days a week for 4 weeks, total 20 sessions	6, 12	MAS, SIS, MAL-AOU, MAL-QOM
A15 (23)	Masiero et al.	Italy	65.60 ± 9.2/66.83 ± 7.9	(10/4)/ (10/6)	NeReBot/EE	14/16	8.342 ± 3.2/10.23 ± 2.4/acute	EG = 40 min RT + 80 min CT/day; CG = 120 min CT/day, both 5 days/w for 5 weeks, total 25 sessions	7	FMA-UE, FMA-SE, FMA-WH, MAS
A16 (34)	Sale et al.	Italy	67.7 ± 14.2/67.7 ± 14.2	(15/11)/ (16/11)	MIT-MANUS/InMotion2/EE	26/27	30 ± 7/subacute	45 min/session/d,5 days/w for 6 weeks, total 30 sessions		FMA-UE
A17 (16)	Hsieh et al.	China, Taiwan	52.34 ± 13.20/54.12 ± 9.98	(11/5)/ (12/4)	Bi-Manu-Track /EE	16/16	717.17 ± 469.69/846.54 ± 580.49/chronic	90–105 min/session/d, 5 days/w for 4 weeks, total 20 sessions		FMA-UE, FMA-SE, FMA-WH, MAL-AOU, MAL-QOM
A18 (24)	Masiero et al.	Italy	72.4 ± 7.1/75.5 ± 4.8	(9/2)/ (8/2)	NeReBot/EE	11/10	10.1 ± 4.5/12.5 ± 5.2/acute	40 min/session,5 days/w for 5 weeks, total 25 sessions	3	FMA-UE, FMA-SE, FMA-WH, MAS
A19 (17)	Lo et al.	USA	66 ± 11/63 ± 12	(47/2)/ (27/1)	MIT–Manus robotic system/EE	49/28	1281.6 ± 1424/2207.2 ± 1780/chronic	60 min/session,3 sessions /w for 12 weeks, total 36 sessions	6, 9	FMA-UE, MAS, SIS
A20 (51)	Housman et al.	USA	54.2 ± 11.9/56.4 ± 12.8	(11/3)/ (7/7)	T-WREX/EXO	14/14	2572.18 ± 2931.37/3421.46 ± 3911.54/chronic	60 min/session,3 sessions/w for 8 ~ 9 weeks, total 24 sessions	6	FMA-UE, GS, MAL-AOU, MAL-QOM
A21 (100)	Volpe et al.	USA	62 ± 3/60 ± 3	(8/3)/ (7/3)	Monark Rehab Trainer™/EE	11/10	1065.4 ± 213.08/1217.6 ± 334.84/chronic	60 min/session,3 sessions/w for 6 weeks, total 18 sessions	3	FMA-SE, FMA-WH, MAS, SIS
A22 (86)	Rosati et al.	Italy	63.4 ± 11.8/67.88 ± 9.9	(7/5)/ (6/6)	NeReBot/EE	12/12	5.1 ± 2.1/5.5 ± 3.2/acute	EG = 20 ~ 25 min/session,2 sessions/day,5 days/w for 4 weeks, total 40 sessions; CG = placebo, unimpaired upper limb was exposed to the robotic for 30 min,2 sessions/w.		FMA-SE, FMA-WH
A23 (85)	Masiero et al.	Italy	63.4 ± 11.8/68.8 ± 10.5	(10/7)/ (11/7)	NeReBot/EE	17/18	<7 days/acute	EG = 20 ~ 30 min/session,2 sessions/d,4 h/w for 5 weeks, total 20–30 sessions; CG (with unimpaired upper limb) = 30 min/session, 2 sessions/w for 5 weeks, total 10 sessions;	3, 8	MAS, FMA-SE, FMA-WH
A24 (101)	Lum et al.	USA	63.2 ± 3.6/65.9 ± 2.4	(12/1)/ (8/6)	MIME/EE	13/14	919.29 ± 188.73/876.67 ± 191.78/chronic	60 min/session, total 24 sessions in 2 months	6	FMA-SE, FMA-WH
A25 (35)	Volpe et al.	USA	62 ± 2/67 ± 2	(16/14)/ (14/12)	MIT-MANUS /EE	30/26	22.5 ± 1.3/26.0 ± 1.4/acute	60 min/session/d,5 days/w, total 25 sessions		FMA-SE, FMA-WH
B1 (25)	Castelli et al.	Italy	68.00 ± 15.17/57.58 ± 13.40	(7/5)/(3/9)	Amadeo/EE	12/12	133.33 ± 49.92/136.98 ± 57.53/subacute	EG = 23 min RT + 22 min CT/session/day, CG = 45 min CT/session/day, both 3 days/week for 4 weeks, total 12 sessions		FMA-UE, MAS, MBI
B2 (36)	Li et al.	China	63.7 ± 9.4/63.6 ± 11.4	(17/3)/ (16/4)	SEM™ Glove/SRG	20/20	70.55 ± 49.37/67.35 ± 47.31/subacute	EG = 20 min RT + 40 min CT/session/day, CG = 20 min TOT+40 min CT/session/day; 5 days/week, for 4 weeks, total 20 sessions		FMA-H, MAS, GS
B3 (37)	Shin et al.	Korea	57.00 ± 12.78/63.69 ± 8.58	(10/10)/ (7/9)	RAPAEL® Smart Glove digital system/SRG	20/16	24.70 ± 16.26/34.00 ± 25.49/subacute	EG = 30 min RT + 30 min CT/session/d, CG = 60 min/session/d, both 5 days/w for 4 weeks, total 20 sessions		FMA-UE, FMA-SE
B4 (44)	Bayındır et al.	Turkey	58.5 ± 9.0/57.7 ± 10.4	(11/5)/ (10/7)	Hand Tutor/SRG	16/17	494.46 ± 791.75/242.80 ± 270.68/chronic	EG = 60 min RT + 180 min CT/ session; CG = 180 min CT/ session, both 2 days/w for 5 weeks, total 10 sessions	3	FMA-UE, GS
B5 (7)	Coskunsu et al.	Turkey	59.9 ± 14.2/70.0 ± 14.0	(4/7)/ (7/2)	Hand of Hope (HOH)/EXO	11/9	<4 weeks /acute	EG = 60 min RT 60 min CT/session/d; CG = 60 min CT/ session/d, both 5 days/w for 3 weeks, total 15 sessions		FMA-SE, MAL-AOU, MAL-QOM
B6 (64)	Singh et al.	India	41.1 ± 12.8/42.7 ± 9.3	19/4	electromechanical robotic-exoskeleton/EXO	12/11	420.07 ± 277.00/313.53 ± 152.20/chronic	45 min/session/d,5 days/w for 4 weeks, total 20 sessions		FMA-UE, MAS, FMA-SE, FMA-WH
B7 (52)	Taravati et al.	Turkey	50.94 ± 17.20/55.75 ± 11.61	(14/3)/ (14/6)	ReoGo™-Motorika /SRG	17/20	333.01 ± 244.13/385.07 ± 256.31/chronic	EG = 30 ~ 45 min RT + 60 min CT/session/d; CG = 60 min CT/session/d, both 5 days/ w for 4 weeks, total 20 sessions		FMA-UE, FMA-H, GS
B8 (102)	Hsu et al.	China Taiwan	55.5 ± 13.4/56.3 ± 16.5		Tenodesis-Induced-Grip Exoskeleton Robot (TIGER)/EXO	17/15	718.38 ± 484.00/1104.97 ± 897.98/chronic	EG = 20 min RT + 20 min CT/session; CG = 40 min CT/session;2 sessions/w for 9 weeks, total 18 sessions	3	FMA-UE, FMA-SE, FMA-H, MAL-AOU, MAL-QOM
B9 (48)	Ranzanic et al.	Switzerland	70.00 ± 12.79/67.46 ± 11.39	(10/4)/ (8/5)	ReHapticKnob/EE	14/13	3.14 ± 1.51/3.08 ± 1.32/acute	EG = 45 min RT + 45 min CT + 30 min CT/ 3 sessions, CG = 2*45 min CT + 30 min CT/ 3 sessions. on 15 days distributed over 4 weeks, total 45 sessions	2, 8	FMA-UE, FMA-SE, FMA-WH, MAS
B10 (38)	Dehem et al.	Belgium	67.3 ± 11.1/68.6 ± 19.1	(11/12)/ (10/12)	REAplan robot/EE	23/22	28.1 ± 4.4/27.5 ± 6.6/acute	45 min/session,4 sessions/w for 9 weeks, total 36 sessions	6	FMA-UE
B11 (26)	Calabrò et al.	Italy	65 ± 3/64 ± 3	(11/14)/ (14/11)	Amadeo™ hand/EE	25/25	chronic	EG = 45 min intensive RT + 180 min intensive CT/session/d; CG = 45 min intensive CT + 180 min intensive CT/session/d, both 5 days/w for 8 weeks, total 40 sessions		FMA-UE
B12 (39)	Villafañe et al.	Italy	67 ± 11/70 ± 12	(11/5)/ (10/6)	Gloreha/SRG	16/16	<3 months/subacute	EG = 30 min RT/session, 3 sessions/w + 60 min CT/session/d; CG = 30 min CT/session, 3 sessions/w + 60 min/session/d, both 5 days/w for 3 weeks, total 9 sessions RT, 15 sessions CT		MAS
B13 (45)	Orihuela-Espina et al.	Mexico	56.22 ± 13.72/55.00 ± 25.78	(5/4)/ (6/2)	robot Amadeus Tyromotion/EE	9/8	74.27 ± 26.79/66.36 ± 38.05/subacute	40 ~ 60 min/session/d, 5 days/w for 8 weeks, total 40 sessions		FMA-H
B14 (27)	Vanoglio et al.	Italy	72 ± 11/73 ± 14	(7/8)/ (7/8)	glove Gloreha Professional/SRG	15/15	15.2 ± 6.8/17.8 ± 7.9/acute	40 min/session/d,5 days/w for 6 weeks, total 30 sessions		GS
B15 (40)	Thielbar et al.	USA	61 ± 12/56 ± 10	(7/4)/ (8/3)	VAEDA glove/SRG	11/11	2891.8 ± 3470.16/1461.12 ± 1430.68/chronic	60 min/session, 3 sessions/w for 6 weeks, total 18 sessions		FMA-UE, FMA-H, GS
B16 (28)	Susanto et al.	China	50.7 ± 9.0/55.1 ± 10.6	(7/2)/ (7/3)	The modified hand exoskeleton robot/EXO	9/10	499.22 ± 176.55/490.08 ± 155.24/chronic	60 min/session,3 ~ 5 sessions/w for 5 weeks, total 20 sessions	6	FMA-UE, FMA-SE, FMA-WH
B17 (41)	Sale et al.	Italy	67.0 ± 12.4/72.56 ± 8.98	(8/3)/ (6/3)	Amadeo Robotic System/EE	11/9	30 ± 7 days/acute	40 min/session,4/5 days/w for 4/5 weeks, total 20 sessions	3	MAS

¿ The follow-up period was measured in months(m).

### 3.2 Methodological quality of included trials

Methodological quality assessment using the Cochrane RoB 2.0 tool classified the 39 included studies as follows: (1) Low risk: *n* = 11 (26.18%, studies A2 (18), A3 (19), A6 (20), A7 (21), A13 (22), A15 (23), A18 (24), B1 (25), B11 (26), B14 (27), B16 (28)); (2) Some concerns: *n* = 26 (61.91%, studies A1 (29), A4 (14), A5 (30), A8 (31)-A9 (32), A11 (33), A16 (34), A17 (16), A19 (17)-A25 (35), B2 (36), B3 (37), B5 (7)-B10 (38), B12 (39), B15 (40), B17 (41)); (3) High risk: *n* = 5 (11.91%, studies A10 (42), A12 (43), A14 (15), B4 (44), B13 (45)).

Key risk determinants included methodological flaws (inadequate allocation concealment documentation and unreported randomization procedures) and implementation issues (absence of blinding protocols and attrition rates >20%).

**Figure 2:**
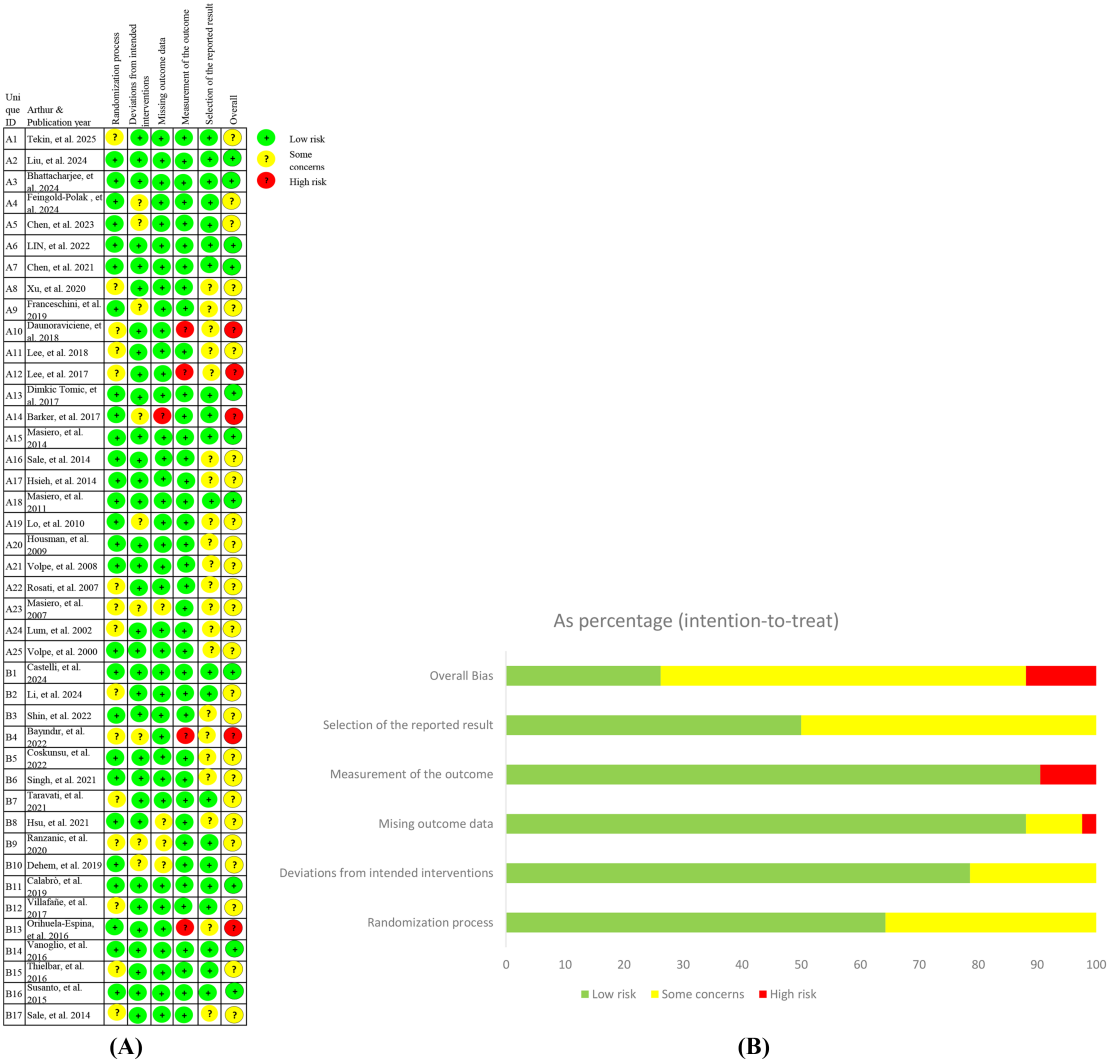
Risk of bias for included studies (ROB 2.0). **(A)** Risk of bias graph; **(B)** Risk of bias summary.

Complete evaluation details are visualized in Figure 2.

### 3.3 Outcome measures

#### 3.3.1 Primary outcome measures

##### 3.3.1.1 FMA-UE

FMA-UE, a validated measure of post-stroke motor recovery and neuromuscular control (46, 47), was analyzed across 30 studies (1,204 participants). Baseline FMA-UE scores demonstrated homogeneity between groups (fixed-effect model: *I*^2^ = 6.90%, Cochran’s Q *p* = 0.359, WMD = 0.20, 95% CI (0.63, 1.03), *p* = 0.640). Post -intervention analysis under a random-effects model (*I*^2^ = 81.90%, Cochran’s Q *p* < 0.001) revealed statistically superior outcomes in robotic-assisted rehabilitation versus conventional therapy (WMD = 6.17, 95% CI (3.81, 8.53), *p* < 0.001).

Subgroup analyses revealed differential efficacy of experimental group (EG) versus conventional group (CG) across stroke chronicity phases. In the acute phase (≤1 month), no significant between-group difference was observed (WMD = 4.09, 95% CI (−2.56, 10.74), *p* = 0.228). However, during the subacute phase (1–6 months), EG demonstrated statistically superior outcomes (WMD = 8.82, 95% CI (4.42, 13.23), *p* < 0.001), with a reduced but persistent effect in the chronic phase (≥6 months; WMD = 3.74, 95% CI (1.81, 5.66), *p* < 0.001). Interaction testing across phases showed no significant subgroup differences (*p* = 0.115).

In subgroup analyses, significant improvements were observed in both age groups: participants aged ≤60 years (WMD = 6.30, 95% CI (3.76, 8.84), *p* < 0.001) and those aged >60 years (WMD = 5.72, 95% CI (1.19, 10.25), *p* = 0.013). However, the difference between these age groups was not statistically significant (*p* = 0.826). Intervention parameters further influenced outcomes: proximal joint-focused therapy yielded greater improvements (WMD = 6.09, 95% CI (3.77, 8.42), *p* < 0.001) compared to distal joint training (WMD = 5.59, 95% CI (0.84, 10.34), *p* = 0.021). Both session length subgroups (≤30 min: WMD = 5.80, 95% CI (2.70, 8.91), *p* < 0.001; >30 min: WMD = 6.34, 95% CI (3.20, 9.51), *p* < 0.001) demonstrated significant within-group improvements, with no evidence of differential effects between subgroups (*p* = 0.807). Significant improvements were observed in both subgroups based on training duration: <24 sessions (WMD = 7.29, 95% CI (3.90, 10.69), *p* < 0.001) and ≥24 sessions (WMD = 2.98, 95% CI (1.50, 4.46), *p* < 0.001). Although the interaction effect between subgroups was not statistically significant (*p* = 0.090), the magnitude of improvement was larger in the <24 sessions subgroup.

Subgroup analysis by robotic type included 29 studies (*n* = 1,184; one study A4 (14) excluded due to unclassifiable robot). Baseline data showed no significant imbalance (fixed-effect model: WMD = 0.18, 95% CI (−0.65, 1.02), *p* = 0.668; *I*^2^ = 9.60%, Cochran’s Q *p* = 0.318). For endpoint outcomes (random-effects model: *I*^2^ = 82.10%, Cochran’s Q *p* < 0.001), the experimental group demonstrated superior overall efficacy (WMD = 5.94, 95% CI (3.55, 8.34), *p* < 0.001). Category-specific effects revealed significant benefits for EXO (WMD = 6.24, 95% CI (2.88, 9.60), *p* < 0.001) and EE (WMD = 6.04, 95% CI (2.17, 9.91), *p* = 0.002), but not for SRG (WMD = 4.33, 95% CI (−0.98, 9.63), *p* = 0.110). No significant heterogeneity was observed across robot categories (*p* = 0.828).

Subgroup analysis of follow-up periods included 11 studies (*n* = 354), with one study (B9 (48), *n* = 27) contributing to both subgroups due to dual timepoint measurements (2/8 months post-treatment). Baseline data showed no significant differences (fixed-effect: WMD = 0.92, 95%CI (−0.62, 2.46), *p* = 0.242; *I*^2^ = 0.00%, Cochran’s Q *p* = 0.593). Significant treatment effects emerged at:

End-of-treatment (fixed-effect: *I*^2^ = 48.90%, Cochran’s Q *p* = 0.034; WMD = 2.05, 95%CI (0.69, 3.40), *p* = 0.003; interaction *p* = 0.893).

Follow-up (random-effects: *I*^2^ = 61.80%, Cochran’s Q p = 0.003; WMD = 5.17, 95%CI (1.75, 8.58), *p* = 0.003; interaction *p* = 0.749).

EG demonstrated sustained efficacy in studies with >3-month follow-up:

End-of-treatment: WMD = 4.04, 95%CI (1.20, 6.88), *p* = 0.005.

Follow-up: WMD = 5.57, 95%CI (2.79, 8.36), *p* < 0.001.

No significant benefits were observed in ≤3-month follow-up studies at either timepoint (end-of-treatment: WMD = 3.47, 95%CI (−0.19, 7.13), *p* = 0.063; follow-up: WMD = 4.50, 95%CI (−0.05, 9.05), *p* = 0.053).

Stratified by baseline impairment severity (49) significant improvements occurred in severe (FMA-UE 0–28: WMD 7.53, 95% CI (2.90, 12.15), *p* = 0.001) and moderate (FMA-UE 29–42: WMD 3.85, 95% CI (2.44, 5.26), *p* < 0.001) subgroups, but not in mild impairment (FMA-UE 43–66: WMD 4.77, 95% CI (−1.25, 10.79), *p* = 0.121). No statistically significant heterogeneity was observed across severity strata (*p* = 0.554).

**Figure 3:**
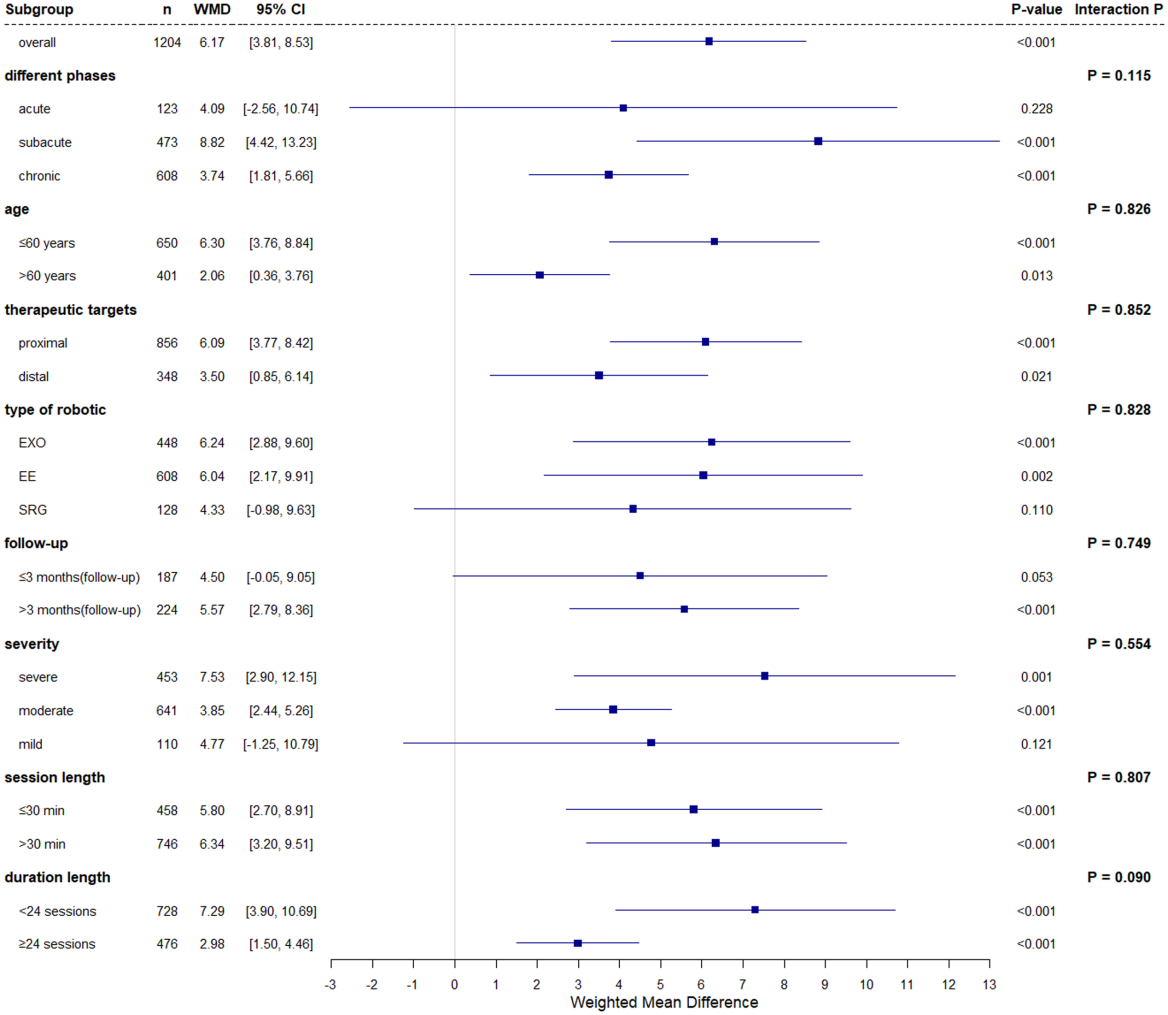
The subgroup analysis forest plot of FMA-UE.

The forest plot results of the FMA-UE subgroup analysis are shown in Figure 3.

##### 3.3.1.2 MAS

MAS were analyzed across 11 RCT comprising 344 participants. Baseline measurements showed no intergroup differences in muscle tone characteristics (WMD = −0.00, 95% CI (−0.00, 0.00), *p* = 1.000). Post-intervention analysis employing a fixed-effect model (*I*^2^ = 17.60%, Cochran’s Q *p* = 0.276) demonstrated non-significant between-group differences in MAS improvement (WMD = −0.08, 95% CI (−0.21, 0.05), *p* = 0.245).

Subgroup analyses identified time-dependent treatment effects, with the EG showing superior outcomes at the first follow-up (WMD = −0.37, 95% CI (−0.66, −0.08), *p* = 0.014) and second follow-up (WMD = −0.46, 95% CI (−0.79, −0.12), *p* = 0.008). Stratification by follow-up duration demonstrated significant EG benefits in the ≤3-month subgroup at both first (WMD = −0.46, 95% CI (−0.77, −0.15), *p* = 0.004) and second follow-ups (WMD = −0.53, 95% CI (−0.88, −0.18), *p* = 0.003), while the follow-up >3-month subgroup retained EG superiority only at the second follow-up (WMD = −0.41, 95% CI (−0.78, −0.04), *p* = 0.032). No significant effects were observed in other prespecified subgroups.

Notably, 2 trials (A15 (23) and B9 (48); *n* = 57) employed dual follow-up assessments encompassing both ≤3-month and >3-month observation periods in their study protocols.

##### 3.3.1.3 GS

Grip strength, a quantitative measure of upper extremity function in stroke patients, serves as both a prognostic indicator for motor recovery in chronic stroke and a correlate of functional capacity (ADL) (50). Analysis of 7 RCTs (*n* = 240) utilizing SMD to account for measurement heterogeneity revealed significant baseline variability across studies (SMD = 0.33, 95% CI (0.07, 0.59), *p* = 0.012; *I*^2^ = 47.90%, Cochran’s Q *p* = 0.073). Post-intervention analysis under a fixed-effect model (*I*^2^ = 34.50%, *p* = 0.165) demonstrated statistically superior grip strength recovery in the robotic intervention group compared to conventional therapy (SMD = 0.45, 95% CI (0.19, 0.71), *p* = 0.001).

Subgroup analyses accounting for baseline heterogeneity revealed significant pretreatment disparities in different phases (SMD = 0.44, 95% CI (0.01, 0.87), *p* = 0.047), chronic stroke patients (SMD = 0.70, 95% CI (0.13, 1.27), *p* = 0.015), age≤60-year-old cohort (SMD = 0.55, 95% CI (0.08, 1.02), *p* = 0.021), and session length<24-session intervention subgroup (SMD = 0.31, 95% CI (0.01, 0.61), *p* = 0.041). Post-treatment analyses demonstrated statistically superior grip strength gains in these subgroups (different phases: SMD = 0.41, 95% CI (0.13, 0.70), *p* = 0.004); chronic: SMD = 0.59, 95% CI (0.21, 0.96), *p* = 0.002; ≤60 years: SMD = 0.49, 95% CI (0.02, 0.95), *p* = 0.041; <24 sessions: SMD = 0.35, 95% CI (0.05, 0.65), *p* = 0.020, though these results may reflect residual confounding from baseline imbalances. Significant therapeutic effects were observed across most subgroups (all *p* < 0.05), with exceptions in acute stroke (SMD = 0.17, 95% CI (−0.27, 0.61), *p* = 0.454), proximal joints (SMD = 0.56, 95% CI (−0.61, 1.74), *p* = 0.348), and ≤30-min session protocols (SMD = 0.30, 95% CI (−0.33, 0.93), *p* = 0.348). The observed subgroup-specific efficacy warrants cautious interpretation given potential confounding inherent in *post hoc* analyses.

##### 3.3.1.4 MBI

The MBI, a globally validated measure of activities of daily living (ADL), was analyzed across 7 studies involving 412 participants. Baseline assessments showed no significant intergroup differences (WMD = 1.01, 95% CI (−2.10, 4.12), *p* = 0.524). Post-intervention analysis using a fixed-effect model (*I*^2^ = 31.80%, Cochran’s Q *p* = 0.185) demonstrated statistically superior ADL improvement in the robotic intervention group compared to conventional therapy (WMD = 8.00, 95% CI (4.96, 11.03), *p* < 0.001).

Exploratory subgroup analyses focusing on proximal upper-limb robotic interventions—limited by small sample sizes across stratification variables (treatment duration, robot type, session frequency, and total training volume)—consistently favored the experimental group (all subgroup *p* < 0.05). These findings suggest robust ADL benefits of robotic rehabilitation, though subgroup interpretations require caution due to underpowered stratification analyses and potential confounding from unmeasured variables.

##### 3.3.1.5 SIS

The SIS, a validated instrument for assessing post-stroke participation capacity, was analyzed across 5 studies involving 196 participants. Baseline comparisons revealed no significant intergroup differences (WMD = 0.13, 95% CI (−2.55, 2.80), *p* = 0.925). Post-intervention analysis using a fixed-effect model (*I*^2^ = 0.00%, Cochran’s Q *p* = 0.459) demonstrated statistically superior participation improvement in the EG compared to CG (WMD = 4.19, 95% CI (1.55, 6.84), *p* = 0.002).

Exploratory subgroup analyses—limited by small sample sizes across stratification variables (stroke phase, device type, and age)—identified significant therapeutic benefits in subacute stroke patients (WMD = 4.15, 95% CI (0.84, 7.47), *p* = 0.014) and the age≤60-year-old cohort (WMD = 4.15, 95% CI (0.84, 7.47), *p* = 0.014). These findings suggest age- and phase-dependent efficacy of robotic rehabilitation, though the limited subgroup granularity necessitates cautious interpretation of differential treatment effects.

#### 3.3.2 Secondary outcome measures

##### 3.3.2.1 FMA-SE

Analysis of the FMA-SE subscale—specifically evaluating proximal upper limb motor function (shoulder, elbow, forearm)—included 17 studies with 640 participants. Baseline comparisons revealed statistically significant intergroup differences favoring the robotic intervention group (WMD = 1.02, 95% CI (0.39, 1.65), Cochran’s Q *p* = 0.002). Post-treatment analysis using a fixed-effect model (*I*^2^ = 43.40%, Cochran’s Q *p* = 0.029) demonstrated sustained between-group disparities (WMD = 2.10, 95% CI (1.17, 3.04), *p* < 0.001). However, the observed therapeutic advantage may reflect residual confounding from pretreatment imbalances rather than true intervention effects, as baseline discrepancies exceeded clinically meaningful thresholds.

##### 3.3.2.2 FMA-WH

The FMA-WH subscale, assessing distal upper extremity motor function (wrist and hand domains), was analyzed across 10 studies involving 273 participants. Baseline comparisons revealed no statistically significant intergroup differences (WMD = 0.00, 95% CI (−0.00, 0.00), *p* = 1.000). Post-intervention analysis using a fixed-effect model (*I*^2^ = 36.90%, Cochran’s *p* = 0.113) demonstrated statistically superior motor recovery in the robotic intervention group compared to conventional therapy (WMD = 1.03, 95% CI (0.69, 1.37), *p* < 0.001). These findings suggest targeted benefits of robotic rehabilitation for distal limb function, though the clinical significance of the observed effect size warrants further investigation given the modest sample size and potential measurement variability in wrist/hand assessments.

##### 3.3.2.3 FMA-H

Analysis of the FMA-H subscale, a dedicated measure of hand-specific motor recovery within the FMA-UE framework, included 6 studies with 196 participants. Baseline assessments showed no significant intergroup differences in hand function (WMD = 1.03, 95% CI (0.01, 2.07), *p* = 0.088). Post-intervention analysis using a fixed-effect model (low heterogeneity: *I*^2^ = 25.80%, Cochran’s *p* = 0.241) revealed statistically superior hand motor improvement in the robotic rehabilitation group compared to conventional therapy (WMD = 2.06, 95% CI (0.97, 3.15), *p* < 0.001). These results demonstrate targeted efficacy of robotic interventions in post-stroke hand rehabilitation, though the clinical relevance of the observed effect size (2.39-point improvement on FMA-H) warrants further validation through larger-scale trials with extended follow-up periods, particularly given the limited sample size and potential ceiling effects inherent in hand-specific functional assessments.

##### 3.3.2.4 MAL-AOU

Analysis of the MAL-AOU subscale—a validated measure of paretic upper limb utilization frequency in real-world settings—encompassed six studies with 166 participants. Baseline comparisons revealed statistically significant intergroup differences favoring the robotic intervention group (WMD = 0.27, 95% CI (0.09, 0.45), *p* = 0.003). Post-treatment analysis under a fixed-effect model (*I*^2^ = 0.00%, *p* = 0.606) demonstrated sustained between-group disparities (WMD = 0.39, 95% CI (0.18, 0.59), *p* < 0.001). However, the magnitude of baseline imbalance (0.27-point advantage on MAL-AOU) raises concerns about residual confounding, suggesting the observed post-intervention differences may partially reflect pretreatment variance rather than therapeutic efficacy.

##### 3.3.2.5 MAL-QOM

Analysis of the MAL-QOM subscale, assessing paretic limb movement quality in real-world contexts, included 6 studies and 166 participants. Baseline assessments revealed statistically significant intergroup differences (WMD = 0.21, 95% CI (0.04, 0.39), *p* = 0.016). Post-intervention analysis employing a fixed-effect model (*I*^2^ = 0.00%, Cochran’s *p* = 0.871) demonstrated sustained between-group disparities (WMD = 0.32, 95% CI (0.11, 0.53), *p* = 0.003). However, the pretreatment advantage of 0.21 MAL-QOM points in the robotic group introduces substantial confounding risk, suggesting these results may reflect baseline imbalances rather than therapeutic intervention effects. The cumulative 0.53-point total difference (baseline + intervention effects) approaches clinically meaningful thresholds for functional recovery, yet definitive conclusions require confirmation through rigorously controlled trials with matched baseline characteristics.

No significant differential treatment effects were observed across pre-specified subgroups (including stroke stage, age, intervention duration, injury severity, and adjunctive parameters). The wide confidence intervals spanning zero in some subgroups suggest clinically non-significant differences or potential type II error due to limited sample size.

Forest plots illustrating all outcome measures across baseline, endpoint, and follow-up assessments are shown in Supplementary Figures S1, S2.

#### 3.3.3 Impact of baseline imbalance and confounding adjustment

Meta-regression analyses were performed to quantify the confounding effects of baseline imbalances on the estimated treatment effects. The results revealed significant associations for several outcome measures:

GS: Baseline imbalance significantly influenced outcomes (*β* = 0.666, 95% CI (0.11, 1.23), *p* = 0.020), primarily attributed to studies A20 (51), B4 (44), B7 (52), and B15 (40). After statistical adjustment for this imbalance, the treatment effect was substantially attenuated and became non-significant (Pooled Hedge’s g = 0.224, 95% CI (−0.10, 0.55), *p* = 0.174), indicating that the unadjusted effect was likely inflated.

GS Subgroups: different phases (*β* = 0.628, *p* = 0.022), Chronic phase (*β* = 0.799, *p* = 0.001), age ≤60 years (*β* = 0.848, *p* = 0.001), and duration <24 sessions (*β* = 0.700, *p* = 0.007) showed strong baseline effects (sources: A20 (51), B15 (40)). Adjusted effects remained non-significant (*p* > 0.05).

MBI (session ≤30 min): Baseline imbalance impacted outcomes (*β* = 1.36, *p* = 0.046), though adjusted effects were non-significant (WMD = −1.24, *p* = 0.493).

MAL Scales (AOU/QOM): Baseline effects were significant (AOU: *β* = 0.62, *p* = 0.028; QOM: *β* = 0.58, *p* = 0.032), largely driven by study A20 (51). Adjusted effects were non-significant (*p* > 0.05).

These results indicate that the apparent benefits of robotic therapy on grip strength and functional activity are likely inflated by pre-existing group differences, and definitive conclusions on its efficacy for these specific outcomes cannot be drawn from the present data.

FMA-SE: In contrast to other measures, the significant treatment effect for FMA-SE demonstrated robustness against baseline confounding (*β* = 0.85, *p* < 0.001), the adjusted treatment effect remained both statistically and clinically significant (Pooled Hedge’s g = 0.92, 95% CI (0.23, 1.61), *p* = 0.009).

This confirms that the improvement in proximal upper limb motor function is a reliable and robust finding, independent of baseline confounding.

Complete statistical outputs for all analyses, including subgroup-specific confidence intervals and source study details, are provided in Supplementary Table S3. Sensitivity analyses further corroborating these findings are presented in Supplementary Tables S3-3–S3-13.

#### 3.3.4 Sensitivity analysis and publication bias

Significant publication bias was detected through Egger’s test in critical analyses, with statistically significant effects observed for the FMA-UE chronic phase subgroup (*p* = 0.044) and age≤60 years subgroup (*p* = 0.002), as well as across all grip strength (GS) analyses including the overall assessment (*p* = 0.012), different phases subgroup (*p* = 0.005), and age≤60 years subgroup (*p* = 0.014). Trim and fill adjustment confirmed the robustness of these findings, as evidenced by the FMA-UE subgroups demonstrating enhanced significance after adjustment (chronic phase: *p* = 0.044 → 0.000; age≤60: *p* = 0.002 → 0.000), while GS subgroups maintained statistical significance post-adjustment (overall: *p* = 0.017; different phases: *p* = 0.030; age≤60: *p* = 0.013). These analyses collectively indicate that although publication bias was statistically evident, trim and fill adjustments consistently demonstrated robust effect estimates across all outcome measures. Complete publication bias statistics, including Egger’s intercept values and trim and fill-adjusted outcomes, are detailed in Supplementary Table S4, with corresponding funnel plots incorporating imputed studies provided in Supplementary Figure S3.

#### 3.3.5 Standardized stroke phase classification

The reclassification protocol was applied to 1,583 participants with available data. The distribution of time from stroke onset yielded a median of 60.5 days (IQR: 28.3–182.7). This empirical distribution was used to calibrate and validate the following predefined phase thresholds: Acute phase: ≤1 month (≤30 days) post-onset, subacute: 1–6 months (31–182 days), and chronic: ≥6 months (≥183 days).

The median (60.5 days) falling within the subacute phase and the IQR upper bound (182.7 days) aligning closely with the chronic threshold supported the validity of this classification for our cohort. Validation confirmed that extreme values (>5 years) exerted negligible influence on the median (weighted simulation error <2%). This reclassification resolves prior definitional heterogeneity, ensuring the validity of subsequent time-stratified analyses. Detailed original and reclassified phases for all studies are provided in Supplementary Table S5.

#### 3.3.6 Sensitivity analysis post high-risk study exclusion

To assess the robustness of our findings against methodological quality concerns, we performed a sensitivity analysis by excluding the five studies judged to be at high overall risk of bias (A10 (42), A12 (43), A14 (15), B4 (44), B13 (45)). The results for the primary outcome, FMA-UE, remained stable and statistically significant (WMD = 5.91, 95% CI (3.45, 8.37), *p* < 0.001), confirming the core finding of this meta-analysis. Similarly, no substantial variations were observed in other primary outcomes, including the clinically meaningful improvement in activities of daily living (MBI).

This exclusion, however, refined our understanding of two secondary outcomes:

- For grip strength (GS), the previously significant baseline heterogeneity became non-significant (5 studies, *n* = 157; SMD = 0.48, 95% CI (−0.07, 1.02), *p* = 0.085).
- For the FMA-Hand (FMA-H) subscale, a significant baseline difference favoring the control groups was revealed after exclusion (5 studies, *n* = 179; SMD = 1.40, 95% CI (0.30, 2.49), *p* = 0.013).

Crucially, despite these shifts in baseline comparability, the endpoint treatment effects for both GS and FMA-H remained stable and statistically significant (GS: SMD = 0.63, 95% CI (0.30, 0.95), *p* < 0.001; FMA-H: SMD = 2.03, 95% CI (0.89, 3.18), *p* = 0.001). This indicates that the efficacy signals for these outcomes are not solely driven by the identified high-risk studies.

Detailed results of this sensitivity analysis are provided in Supplementary Table S6.

Subgroup analyses were performed, and comprehensive statistical analyses were conducted for all primary and secondary outcome measures, with the detailed results presented in Table 2.

**Table 2:** Subgroup analysis and detailed data analysis results of the outcome measures.

Outcome	Categories	Studies	Participants	Baseline				End of treatment				*p*-value between sub-group (DL subgroup weights)	Publication bias (Egger’s Test)	Trim & Fill
				Heterogeneity		WMD (95%CI)	*p*-value	Heterogeneity		WMD (95%CI)	*p-*value			
				*I* ^2^	*p-*value (Cochran’s Q)			*I* ^2^	*p-*value (Cochran’s Q)					
FMA-UE	All studies	30	1,204	6.90%	0.359	0.20 (−0.63, 1.03)	0.640	81.90%	0.000	6.17 (3.81, 8.53)	0.000		0.222	
	Different phases													
	Acute	4	123	0.00%	0.683	2.60 (−4.70, 9.90)	0.486	0.00%	0.553	4.09 (−2.56, 10.74)	0.228	0.115	0.160	
	Subacute	12	473	0.00%	0.600	0.11 (−1.20, 1.41)	0.873	86.40%	0.000	8.82 (4.42, 13.23)	0.000		0.773	
	Chronic	14	608	34.90%	0.096	0.21 (−0.88, 1.30)	0.707	50.50%	0.016	3.74 (1.81, 5.66)	0.000		**0.044** §§	0.004
	Age													
	≤60 years	16	650	0.00%	0.504	1.22 (−0.03, 2.47)	0.055	72.70%	0.000	6.30 (3.76, 8.84)	0.000	0.826	**0.002** §§	0.000
	>60 years	14	554	0.00%	0.279	−0.62 (−1.73, 0.50)	0.228	86.50%	0.000	5.72 (1.19, 10.25)	0.013		0.766	
	Therapeutic targets													
	Proximal	19	856	4.00%	0.408	0.83 (−0.53, 2.19)	0.231	58.90%	0.001	6.09 (3.77, 8.42)	0.000	0.852	0.059	
	Distal	11	348	9.70%	0.352	−0.18 (−1.23, 0.87)	0.739	91.40%	0.000	5.59 (0.84, 10.34)	0.021		0.710	
	Type of robotic‡	29	1,184	9.60%	0.318	0.18 (−0.65, 1.02)	0.668	82.10%	0.000	5.94 (3.55, 8.34)	0.000		0.280	
	EXO	9	448	5.00%	0.394	1.61 (−0.16, 3.37)	0.074	67.20%	0.002	6.24 (2.88, 9.60)	0.000	0.828	0.231	
	EE	16	608	0.00%	0.620	−0.53 (−1.60, 0.54)	0.331	84.90%	0.000	6.04 (2.17, 9.91)	0.002		0.230	
	SRG	4	128	41.40%	0.163	0.89 (−1.17, 2.95)	0.398	67.10%	0.028	4.33 (−0.98, 9.63)	0.110		0.245	
	Follow-up§	11^a^ ¶	354^a^ ¶	0.00%	0.593	0.92 (−0.62, 2.46)	0.242	48.90%	0.034	2.05 (0.69, 3.40)	0.003		0.135	
	≤3 months§	6	187	5.20%	0.383	0.47 (−1.27, 2.20)	0.599	57.20%	0.039	3.47 (−0.19, 7.13)	0.063	0.893	0.083	
	>3 months§	7	224	0.00%	0.803	2.53 (−0.59, 5.64)	0.111	15.60%	0.311	4.04 (1.20, 6.88)	0.005		0.682	
	Follow-up1’§	11^a^ ¶	354^a^ ¶	0.00%	0.593	0.92 (−0.62, 2.46)	0.242	61.80%	0.003	5.17 (1.75, 8.58)	0.003		0.135	
	≤3 m ‘§	6	187	5.20%	0.383	0.47 (−1.27, 2.20)	0.599	67.00%	0.010	4.50 (−0.05, 9.05)	0.053	0.749	0.202	
	>3 m ‘§	7	224	0.00%	0.803	2.53 (−0.59, 5.64)	0.111	25.80%	0.232	5.57 (2.79, 8.36)	0.000		0.990	
	Severity													
	Severe	11	453	18.90%	0.264	0.03 (−1.31, 1.36)	0.968	88.30%	0.000	7.53 (2.90, 12.15)	0.001	0.554	0.502	
	Moderate	15	641	23.30%	0.196	0.33 (−0.92, 1.57)	0.607	28.40%	0.145	3.85 (2.44, 5.26)	0.000		0.053	
	Mild	4	110	0.00%	0.927	0.25 (−1.79, 2.30)	0.808	76.20%	0.006	4.77 (−1.25, 10.79)	0.121		0.261	
	Session length													
	≤30 min	9	458	35.70%	0.133	0.89 (−0.91, 2.66)	0.335	61.00%	0.009	5.80 (2.70, 8.91)	0.000	0.807	0.356	
	>30 min	21	746	0.00%	0.588	0.01 (−0.93, 0.95)	0.984	85.60%	0.000	6.34 (3.20, 9.51)	0.000		0.354	
	Duration length													
	<24 sessions	18	728	0.00%	0.507	0.47 (−0.61, 1.54)	0.396	87.10%	0.000	7.29 (3.90, 10.69)	0.000	0.090	0.350	
	≥24 sessions	12	476	23.10%	0.217	−0.20 (−1.51, 1.11)	0.765	41.50%	0.065	2.98 (1.50, 4.46)	0.000		0.217	
MAS	All studies	11	344	0.00%	0.450	−0.00 (−0.00, 0.00)	1.000	17.60%	0.276	−0.08 (−0.21, 0.05)	0.245		0.639	

	Different phases													
	Acute	5	133	10.80%	0.344	−0.00 (−0.00, 0.00)	1.000	51.30%	0.084	0.08 (−0.39, 0.54)	0.736	0.380	0.291	
	Subacute	3	90	2.10%	0.360	0.02 (−0.14, 0.19)	0.778	0.00%	0.595	−0.04 (−0.22, 0.15)	0.696		0.393	
	Chronic	3	121	10.00%	0.329	−0.12 (−0.35, 0.11)	0.303	0.00%	0.614	−0.23 (−0.48, 0.03)	0.078		0.375	
	Age													
	≤60 years	2	57	61.10%	0.109	0.15 (−0.55, 0.86)	0.668	0.00%	0.431	−0.23 (−0.59, 0.12)	0.199	0.354	N/A	
	>60 years	9	287	0.00%	0.504	−0.00 (−0.00, 0.00)	1.000	24.90%	0.222	−0.05 (−0.19, 0.09)	0.454		0.785	
	Therapeutic targets													
	Proximal	6	218	2.90%	0.398	−0.00 (−0.00, 0.00)	1.000	22.70%	0.263	−0.08 (−0.28, 0.12)	0.453	0.993	0.398	
	Distal	5	126	13.80%	0.326	−0.02 (−0.17, 0.13)	0.754	29.30%	0.226	−0.08 (−0.25, 0.09)	0.374		0.183	
	Follow-up§	7^b^ **	188^b^ **	26.10%	0.230	0.00 (−0.00, 0.00)	1.000	31.60%	0.187	0.02 (−0.23, 0.27)	0.879		0.150	
	≤3 m§	5	134	0.00%	0.636	−0.00 (−0.00, 0.00)	1.000	36.20%	0.180	−0.00 (−0.26, 0.26)	0.977	0.275	0.452	
	>3 m§	3	91	32.30%	0.228	0.00 (−0.00, 0.00)	1.000	0.00%	0.392	−0.25 (−0.60, 0.11)	0.169		0.091	
	Follow-up1’§	5^b^ **	147^b^ **	0.00%	0.531	−0.00 (−0.00, 0.00)	1.000	24.20%	0.260	−0.37 (−0.66, −0.08)	0.014		0.109	
	≤3 m’§	4	113	0.00%	0.791	−0.00 (−0.00, 0.00)	1.000	0.00%	0.538	−0.46 (−0.77, −0.15)	0.004	0.512	0.125	
	>3 m’§	3	91	32.30%	0.228	−0.00 (−0.00, 0.00)	1.000	57.80%	0.093	−0.18 (−0.97, 0.61)	0.658		0.428	
Follow-up2”§:	4^b^ **	126^b^ **	0.00%	0.393	0.00 (−0.00, 0.00)	1.000	0.00%	0.482	−0.46 (−0.79, −0.12)	0.008		0.519	
GS‡‡ MBI
	≤3 m”§	3	92	0.00%	0.647	−0.00 (−0.00, 0.00)	1.000	0.00%	0.899	−0.53 (−0.88, −0.18)	0.003	0.636	0.956	
	>3 m”§	3	91	32.30%	0.228	0.00 (−0.00, 0.00)	1.000	3.80%	0.354	−0.41 (−0, 78, −0.04)	0.032		0.516	
	Severity	7	231	0.00%	0.745	0.00 (−0.00, 0.00)	1.000	18.70%	0.287	−0.11 (−0.24, 0.03)	0.117		0.612	
	Severe	4	157	0.00%	0.443	−0.04 (−0.25, 0.16)	0.677	0.00%	0.761	−0.10 (−0.27, 0.07)	0.257	0.900	0.325	
	Moderate	3	74	0.00%	0.728	0.00 (−0.00, 0.00)	1.000	67.50%	0.046	−0.13 (−0.53, 0.28)	0.543		0.284	
	Session length													
	≤30 min	2	67	0.00%	0.852	0.01 (−0.19, 0.20)	0.948	0.00%	0.756	0.15 (−0.27, 0.57)	0.481	0.264	N/A	
	>30 min	9	278	18.80%	0.276	−0.00 (−0.00, 0.00)	1.000	25.80%	0.214	−0.10 (−0.24, 0.04)	0.147		0.687	
	Duration length													
	<24 sessions	6	154	33.50%	0.185	0.01 (−0.14, 0.16)	0.876	12.40%	0.336	−0.07 (−0.24, 0.09)	0.390	0.951	0.131	
	≥24 sessions	5	190	0.00%	0.672	−0.00 (−0.00, 0.00)	1.000	37.70%	0.170	−0.08 (−0.29, 0.12)	0.432		0.152		All studies	7	240	47.90%	0.073	0.33 (0.07, 0.59) *	***0.012*** ††	34.50%	0.165	0.45 (0.19, 0.71) *	0.001		**0.012** §§	0.017

	Different phases	6	200	54.80%	0.050	0.44 (0.01, 0.87) *	***0.047*** ††	42.90%	0.119	0.41 (0.13, 0.70) *	0.004		**0.005** §§	0.030
	Acute	2	80	0.00%	0.558	0.03 (−0.41, 0.47) *	0.904	0.00%	0.326	0.17 (−0.27, 0.61) *	0.454	0.155	N/A	
	Chronic	4	120	54.50%	0.086	0.70 (0.13, 1.27) *	***0.015*** ††	48.00%	0.124	0.59 (0.21, 0.96) *	0.002		0.064	
	Age													
≤60 years	5	170	54.00%	0.069	0.55 (0.08, 1.02) *	***0.021***††	54.20%	0.068	0.49 (0.02, 0.95) *	0.041	0.828	**0.014** §§	0.013

	>60 years	2	70	0.00%	0.568	0.02 (−0.45, 0.49) *	0.949	0.00%	0.711	0.56 (0.08, 1.04) *	0.022		N/A	
	Therapeutic targets													
	Proximal	2	78	72.70%	0.055	0.56 (−0.37, 1.49) *	0.238	82.40%	0.017	0.56 (−0.61, 1.74) *	0.348	0.449	N/A	
	Distal	5	162	47.00%	0.110	0.28 (−0.03, 0.60) *	0.081	0.00%	0.505	0.48 (0.17, 0.80) *	0.003		0.160	
	Session length													
	≤30 min	2	90	0.00%	0.991	0.13 (−0.28, 0.55) *	0.535	54.70%	0.138	0.30 (−0.33, 0.93) *	0.348	0.449	N/A	
	>30 min	5	150	60.20%	0.040	0.53 (−0.00, 1.06) *	0.052	31.80%	0.210	0.56 (0.23, 0.89) *	0.001		0.084	
	Duration length													
	<24 sessions	5	182	38.10%	0.167	0.31 (0.01, 0.61) *	***0.041***††	26.80%	0.243	0.35 (0.05, 0.65) *	0.020	0.292	0.070	
	≥24 sessions	2	58	79.90%	0.026	0.46 (−0.74, 1.65) *	0.456	43.80%	0.182	0.79 (0.24, 1.33) *	0.004		N/A	
	All studies	7	412	49.80%	0.063	1.01 (−2.10, 4.12)	0.524	31.80%	0.185	8.00 (4.96, 11.03)	0.000		0.514	
	Different phases													
	Subacute	5	212	7.00%	0.367	−1.86 (−5.58, 1.86)	0.327	50.50%	0.089	6.97 (1.35, 12.59)	0.015	0.571	0.137	
	Chronic	2	200	0.00%	0.789	7.67 (2.00, 13.34)	0.008	0.00%	0.529	9.12 (4.22, 14.02)	0.000		N/A	
	Type of robotic													
	EXO	3	270	42.60%	0.175	2.13 (−4.17, 8.42)	0.508	0.00%	0.861	7.65 (3.18, 12.11)	0.001	0.953	0.480	
	EE	4	142	61.60%	0.050	0.87 (−5.97, 7.71)	0.803	64.50%	0.037	7.90 (0.80, 15.00)	0.029		0.645	
Session length													

	≤30 min	4	288	0.00%	0.586	5.16 (0.90, 9.42)	***0.018*** ††	21.50%	0.282	6.27 (2.41, 10.13)	0.001	0.155	0.593	
	>30 min	3	124	10.20%	0.328	−3.73 (−8.28, 0.83)	0.109	32.40%	0.228	10.81 (5.88, 15.74)	0.000		0.464	
	Duration length													
	<24 sessions	5	342	55.50%	0.061	0.17 (−5.15, 5.48)	0.951	42.40%	0.139	8.01 (4.57, 11.44)	0.000	0.990	0.712	
≥24 sessions	2	70	28.00%	0.239	4.87 (−1.91, 11.65)	0.159	46.10%	0.173	7.96 (1.45, 14.47)	0.017		N/A	
SIS	All studies	5	196	29.20%	0.227	0.13 (−2.55, 2.80)	0.925	0.00%	0.459	4.19 (1.55, 6.84)	0.002		0.672	
	Different phases													
	Subacute	3	98	62.00%	0.072	4.34 (−4.75, 13.25)	0.340	0.00%	0.761	4.15 (0.84, 7.47)	0.014	0.916	0.234	
	Chronic	2	98	0.00%	0.598	−0.60 (−2.76, 1.55)	0.583	67.50%	0.079	4.61 (−3.17, 12.38)	0.245		N/A	
	Age													
	≤60 years	3	98	62.00%	0.072	4.34 (−4.57, 13.25)	0.340	0.00%	0.761	4.15 (0.84, 7.47)	0.014	0.916	0.234	
	>60 years	2	98	0.00%	0.598	−0.60 (−2.76, 1.55)	0.583	67.50%	0.079	4.61 (−3.17, 12.38)	0.245		N/A	
FMA-SE	All studies	17	640	0.00%	0.815	1.02 (0.39, 1.65)	***0.002***††	43.40%	0.029	2.10 (1.17, 3.04)	0.000		0.977	
FMA-WH	All studies	10	273	36.90%	0.113	0.00 (−0.00, 0, 00)	1.000	0.00%	0.820	1.03 (0.69, 1.37)	0.000		0.346	
FMA-H	All studies	6	196	47.90%	0.088	1.03 (−0.01, 2.07)	0.051	25.80%	0.241	2.06 (0.97, 3.15)	0.000		0.317	
MAL-AOU	All studies	6	166	0.00%	0.539	0.27 (0.09, 0.45)	***0.003***††	0.00%	0.606	0.39 (0.18, 0.59)	0.000		0.530	
MAL-QOM	All studies	6	166	0.00%	0.693	0.21 (0.04, 0.39)	***0.016***††	0.00%	0.871	0.32 (0.11, 0.53)	0.003		0.621	

‡Study A4 (14) was excluded from robotic subgroup analysis of FMA-UE due to unclassifiable robotic device specifications.

§Follow-up subgroup notation: endpoint data (no symbol, e.g., ≤3 m) = post-training assessment; single prime symbol (e.g., ≤3 m’) = first follow-up; double prime symbol (e.g., ≤3 m”) = second follow-up.

¶ In FMA-UE follow-up analysis: “a” indicates Study B9 (48) reported overlapping data for ≤3-month and >3-month follow-ups (*n* = 27).

**In MAS follow-up analysis: “b” denotes Studies A15 (23) and B9 (48) contributed overlapping data for ≤3-month and >3-month follow-ups (*n* = 57).

††Baseline *p*-values in bold italics indicate significant between-group differences (*p* < 0.05).

‡‡GS outcome (marked with *) used SMD due to methodological heterogeneity in measurement.

§§Bold values in Publication bias column signify significant publication bias (*p* < 0.05).

### 3.4 Synthesis of primary findings

This meta-analysis demonstrated that robot-assisted therapy significantly improves upper limb function post-stroke compared to conventional rehabilitation. Key findings are summarized in Table 3. Overall motor recovery (FMA-UE) was significantly greater in the robot-assisted group (WMD = 6.17, 95% CI (3.81, 8.53)), with the subacute phase subgroup achieving a clinically meaningful improvement (WMD = 8.82). Significant motor benefits were also observed in patients with severe impairment (WMD = 7.53). For functional independence, robot-assisted therapy provided a statistically significant and clinically meaningful gain in activities of daily living (MBI: WMD = 8.00). While social participation (SIS) showed a statistically significant improvement (WMD = 4.19), it did not reach the threshold for clinical meaningfulness. Grip strength improved significantly (SMD = 0.45), though caution is warranted in interpretation due to baseline imbalances. Subgroup analyses confirmed the benefits were most evident in subacute patients, those with moderate-to-severe impairment, and users of exoskeleton or end-effector devices, while being consistent across different training parameters.

**Table 3:** Summary of key meta-analysis findings for primary outcomes.

Outcome measure	Subgroup or overall analysis	No. of included studies (Participants)	WMD/SMD (95% CI)	*I* ^2^	MCID	MCID achieved?	Clinical interpretation
FMA-UE	overall	30 (*n* = 1,204)	6.17 (3.81, 8.53)	81.90%	6.6–10 points in subacute	Mixed	Statistically and clinically superior in key subgroups
	Subacute Phase	12 (*n* = 473)	**8.82 (4.42, 13.23)**	86.40%		Yes**	Robust, clinically meaningful benefit
	Severe Impairment (in Subacute Phase)	11 (*n* = 453)	**7.53 (2.90, 12.15)**	88.30%		Yes**	Primary target population for benefit
MBI	Overall	7 (*n* = 412)	**8.00 (4.96, 11.03)**	31.80%	1.85 points	Yes**	Clinically meaningful improvement in ADL
SIS	Overall	5 (*n* = 196)	4.19 (1.55, 6.84)	0.00%	5.9 points for SIS-ADL domain	No	Statistically significant improvement in participation
Grip Strength (GS)	Overall	7 (*n* = 240)	0.45 (0.19, 0.71) *	34.50%	/	/	Statistically superior, but interpretation confounded by significant baseline imbalance

* Effect size expressed as SMD due to heterogeneity in measurement methods.

** A “Yes” indicates that the point estimate of the WMD meets or exceeds the lower bound of the Minimum Clinically Important Difference (MCID) range.Bolded values in the WMD/SMD column denote point estimates that reached or surpassed the lower bound of the MCID.

## 4 Discussion

### 4.1 Summary and analysis

This study evaluated robotic-assisted upper extremity therapy through the International Classification of Functioning, Disability and Health (ICF) framework established by the World Health Organization (WHO). Our meta-analysis indicates that the benefits of robotic-assisted therapy are not uniform across all stroke patients or outcome measures. By employing a stratified approach, we moved beyond establishing mere statistical superiority to identify the patient groups and conditions under which these benefits are most clinically meaningful. We found that robotic-assisted therapy significantly improves body functions (motor recovery, grip strength), activities (daily living), and participation, while being non-inferior to conventional therapy in managing body structures (spasticity). The most compelling evidence emerged for patients in the subacute phase and those with severe baseline impairment, who achieved improvements in motor function (FMA-UE) and activities of daily living (MBI) that surpassed established MCID thresholds. This supports a move toward a stratified rehabilitation model, where prioritizing resource-intensive robotic interventions for these target populations may help maximize functional independence.

Our findings regarding the differential efficacy across patient subgroups arrive at a pivotal time for clinical practice. Recent clinical guidelines from several authoritative bodies, including the World Stroke Organization (WSO, 2023), the European Stroke Organization (ESO, 2023), and the United States Department of Veterans Affairs and Department of Defense (US VA/DoD, 2025), have included upper-limb robotic therapy as a recommended adjunct to conventional rehabilitation for stroke (53–55). However, a persistent challenge in implementing these recommendations is the insufficient evidence regarding which specific patient populations benefit most from this intervention.

This study sought to address this evidence gap by examining a stratified approach to robotic therapy. Our analysis indicated that patients in the subacute phase (1–6 months post-stroke) with severe upper-limb impairment (FMA-UE: 0–28 points) demonstrated statistically significant and clinically meaningful improvements in both motor function and activities of daily living. These findings suggest that stratification based on stroke chronicity and baseline motor impairment may represent a viable strategy for optimizing rehabilitation outcomes and resource utilization.

Therefore, while supporting the potential utility of robotic therapy within stroke rehabilitation protocols, our study provides an initial framework for its more targeted implementation. The proposed stratification approach offers a methodological foundation for advancing from generalized recommendations toward more individualized treatment strategies in neurorehabilitation practice.

#### 4.1.1 Interpretive context of subgroup analyses

The interpretation of our extensive subgroup analyses should be framed within their methodological limitations. These analyses were exploratory and not pre-powered for interaction effects. The multiplicity of statistical tests increases the risk of Type I error, and thus these subgroup findings should be considered hypothesis-generating rather than confirmatory. We advise caution in drawing clinical conclusions from them alone.

#### 4.1.2 Clinical meaningfulness vs. statistical significance

An important consideration is the distinction between statistical significance and clinical relevance. Our analysis suggests that robotic-assisted therapy may yield not only statistically superior gains but also clinically meaningful improvements in certain domains. The most compelling evidence emerged for motor recovery in the subacute phase, where the improvement (FMA-UE WMD = 8.82) surpassed the lower bound of the established MCID range (6.6–10 points) (56–61), indicating a change likely perceptible to patients. Similarly, the effect on activities of daily living across the cohort (MBI WMD = 8.00) substantially exceeded its MCID threshold (1.85 points) (21), suggesting a high probability for a meaningful enhancement in functional independence. In contrast, while motor improvements in the chronic phase were statistically significant (FMA-UE WMD = 3.74), the effect size fell below the MCID range for this population (4.25–7.25 points) (10), implying that the average benefit may not constitute a substantial functional shift. Likewise, the improvement in social participation (SIS WMD = 4.19), though statistically significant, did not reach the MCID for the participation domain (5.9 points) (62). This gradation of findings underscores that the intervention’s capacity to elicit patient-important changes is not uniform but is most strongly supported for improving functional independence in subacute and more severely impaired patients.

#### 4.1.3 Differential efficacy and neurobiological correlates across recovery phases

Our findings address a critical gap in previous meta-analyses that focused predominantly on single-phase stroke rehabilitation, by systematically evaluating robotic interventions across acute, subacute, and chronic post-stroke phases. Our results delineate a distinct efficacy profile: the most substantial benefits were observed during the subacute phase, a period of heightened neuroplastic potential, while a more modest yet statistically significant effect was maintained in the chronic phase, consistent with prior findings (63). Acute-phase analyses revealed no EG-CG differences (*p* > 0.05), likely attributable to confounding factors including clinical instability, treatment intolerance, heterogeneous spontaneous recovery rates, and limited sample size (7, 24).

Mechanistically, robotic rehabilitation enhances upper limb functional recovery through neuroplastic modulation. Evidence from neuroimaging and electrophysiological studies indicates that robotic training augments proprioceptive-driven cortical excitability in sensorimotor regions (64, 65), strengthens contralateral frontoparietal connectivity (26, 64), and reduces transcallosal inhibition to restore interhemispheric balance (66). These effects align with animal models demonstrating critical post-stroke recovery windows characterized by heightened neural circuit plasticity, particularly within 12 weeks post-onset (67, 68). While maximal functional gains occur in acute (≤7 days) and subacute (7 days–3 months) phases (69–72), our data corroborate Mazzoleni et al. (67) in showing that chronic patients (≥1 year post-stroke) still achieve incremental benefits from intensive robotic therapy, underscoring its utility across disease stages.

#### 4.1.4 Differential response by impairment severity

Our stratified analysis revealed differential therapeutic responses across injury severities. Patients with severe upper limb impairment demonstrated clinically meaningful gains from robotic rehabilitation, corroborating findings by Wu and Klamroth-Marganska et al. (73–75). This may be attributed to enhanced neuroplasticity mechanisms and greater potential for spontaneous recovery in this population, coupled with higher adherence to repetitive robotic training protocols. Conversely, no significant intergroup differences were observed in mildly impaired patients (EG vs. CG, *p* > 0.05), likely influenced by methodological limitations: (1) restricted sample size in mild-injury subgroups; (2) ceiling effects of conventional assessment scales; and (3) prioritized rehabilitation goals focusing on fine motor control and participation metrics, which are less targeted by current robotic systems (48).

#### 4.1.5 Differential efficacy by robotic device type

Robotic rehabilitation systems are classified as exoskeletons (EXO), end-effectors (EE), or soft robotic gloves (SRG), with SRGs categorized separately due to their structural incompatibility with traditional EXO/EE designs (76, 77). Both EXO and EE devices demonstrated statistically and clinically significant improvements in upper limb function compared to traditional therapies (*p* < 0.01), with no significant difference observed between these two modalities (*p* = 0.939), consistent with prior comparative studies (6, 78). In contrast, our analysis found SRGs did not exhibit superiority over traditional methods (*p* = 0.110), diverging from earlier meta-analyses (10, 40). However, this SRG result warrants careful interpretation given the limited included studies (*n* = 4: B3 (37), B4 (44), B7 (52), B15 (40)), small sample size (*n* = 128 total), strict inclusion criteria (excluding hybrid devices), and participant characteristics (predominantly subacute/chronic stage, mild/moderate impairment) coupled with relatively short intervention durations (<24 sessions). Furthermore, as the Fugl-Meyer Assessment for Upper Extremity (FMA-UE) evaluates overall upper limb function, the specific hand-focused training provided by the SRGs in the included studies, potentially delivered at lower intensity or duration compared to EXO/EE protocols, may limit the detection of their effects on this scale. Consequently, the efficacy of SRGs requires further investigation with larger, more targeted studies.

#### 4.1.6 Limited influence of chronological age

Our findings do not provide compelling evidence to support the use of chronological age as a key stratification factor for robotic therapy. The absence of significant subgroup differences indicates that treatment efficacy is largely comparable across age groups. Any observed marginal benefit in younger patients is likely attributable to confounding factors correlated with age—such as overall health status, frailty, or therapy adherence—rather than to age itself (79, 80). This suggests that future research should focus on these specific aging-related factors rather than on chronological age alone to better understand differential treatment responses.

#### 4.1.7 Effects of treatment parameters and long-term sustainability

Our analysis demonstrates that robotic rehabilitation therapy elicited significant functional improvements in both proximal (shoulder/elbow) and distal (wrist/hand) joints in stroke patients. The magnitude of improvement did not differ significantly between joint groups (*p* > 0.05), corroborating findings by Wu et al. (73). This functional equivalence may stem from distinct neural substrates: proximal control primarily engages the corticoreticulospinal tract, whereas distal control relies predominantly on the corticospinal tract (81). Although a marginal advantage for proximal training was observed, this difference was not statistically significant. We hypothesize that the observed functional balance may result from distal training enhancing inter-joint coordination and inducing compensatory proximal stabilization during movement execution (82, 83).

Our subgroup analysis of robot-assisted rehabilitation therapy did not show a significant dose-dependent therapeutic effect. The grouping of training duration (≤ 30 min vs. > 30 min per session) and the classification of cumulative frequency (< 24 sessions vs. ≥ 24 sessions) (10, 84) consistently indicated that the upper limb function improved significantly after the intervention (*p* < 0.001). Consequently, we provisionally recommend prioritizing high-dose protocols (>30 min/session, ≥24 sessions) for moderate-to-severe impairments to optimize functional gains, while considering the minimal effective dose (e.g., 24 sessions × 30 min) for mild cases to enhance resource utilization efficiency. These clinical interpretations require validation through rigorously designed trials.

Longitudinal follow-up data showed sustained functional gains in patients assessed >3 months post-intervention, with progressive improvements observed between treatment completion and final follow-up (85). This delayed enhancement may reflect residual neuroplastic effects from robotic therapy, potentially mediated by long-term cortical reorganization (86). In contrast, patients evaluated ≤3 months post-treatment maintained but did not significantly exceed their immediate post-training functional levels (*p* > 0.05). The attenuated recovery trajectory in shorter follow-up cohorts likely results from reduced post-protocol training intensity and physiological plateaus in neural repair processes.

#### 4.1.8 Limited impact on spasticity

Our analysis demonstrated comparable spasticity modulation between robotic and conventional therapies, with both groups showing modest reductions in MAS scores lacking clinical significance (ΔMAS <1.0 point; *p* > 0.05) (87–89). This aligns with neuroanatomical evidence implicating reticulospinal tract hyperexcitability as the primary driver of post-stroke spasticity (90, 91). Robotic interventions, while effective in enhancing corticospinal pathway plasticity, may inadequately modulate brainstem-mediated reticulospinal circuits critical for stretch reflex regulation (92). Notably, longitudinal assessments confirmed peak spasticity severity at 1–3 months post-stroke (93), suggesting this phase represents a critical window for targeted antispastic interventions.

#### 4.1.9 Uncertain efficacy for grip strength and training considerations

Regarding grip strength outcomes, baseline heterogeneity limited definitive conclusions, though chronic-phase patients exhibited statistically meaningful improvements in the experimental group (EG). This aligns with evidence that chronic-stage functional recovery correlates with affected hemisphere sensorimotor cortex reactivation during grasping tasks (36, 49). Subgroup analyses indicated distal-focused robotic training (>30 min/session) produced superior grip strength gains (*p* = 0.003 vs. proximal training), likely through enhanced proprioceptive feedback to cortical hand representation areas. These findings advocate for task-specific, intensity-optimized robotic protocols in chronic stroke rehabilitation.

#### 4.1.10 Differential effects on activity and participation

A key distinction emerges between the effects of robotic therapy on basic activities of daily living versus broader social participation. The intervention demonstrated a robust and clinically meaningful impact on functional independence (MBI), a finding that underscores its success in restoring foundational self-care capabilities. This benefit is likely mediated by robotic training’s capacity to deliver high-intensity, task-oriented practice, which promotes adaptive neural plasticity in sensorimotor networks and enhances interhemispheric connectivity (26). These neuroplastic mechanisms appear highly effective for recovering the discrete motor functions required for basic activities.

In contrast, the transfer of these gains to enhanced social participation (SIS) was limited and fell below the threshold for clinical importance. This discrepancy suggests that the neuroplastic changes sufficient for improving discrete motor tasks do not automatically translate into the broader cognitive, psychosocial, and environmental adaptations required for successful community reintegration. While a marginal, age-related benefit in participation was observed, its interpretation remains speculative and warrants further investigation to disentangle neurobiological from psychosocial influences. Future interventions may need to explicitly incorporate participation-focused strategies to bridge this gap between motor recovery and real-world involvement.

#### 4.1.11 Kinematic indicators of neurobiological recovery

Although the methodological heterogeneity of kinematic data precluded formal meta-analysis, a narrative synthesis of the included studies offers valuable insights into motor control quality that extend beyond conventional functional scales. The available evidence suggests that robotic devices provide sensitive kinematic measures—such as movement smoothness and reduced muscle co-contraction—that serve as objective biomarkers of neuromuscular improvement (8, 19, 94, 95). These parameters appear to capture the dissolution of pathological synergies and the restoration of inter-joint coordination, changes that may reflect fundamental neurobiological recovery (67, 96). Notably, the observed dissociation between these qualitative motor improvements and functional scores in some studies is particularly instructive; it indicates that kinematics may uniquely quantify the reduction of compensatory strategies, thereby providing a purer measure of true neurological restoration. This perspective is further supported by correlative neurophysiological evidence of normalized interhemispheric connectivity (4, 84, 97). Consequently, while quantitative synthesis was not feasible, the consistent reporting of these kinematic gains across studies underscores their complementary value in elucidating recovery mechanisms and highlights the importance of standardizing these measures in future trials.

#### 4.1.12 Robustness of findings and sensitivity analyses following exclusion of high-risk studies

The sensitivity analysis excluding five high-risk studies (A10 (42), A12 (43), A14 (15), B4 (44), B13 (45)) confirmed the robustness of our primary findings. Treatment effects for the FMA-UE and activities of daily living (MBI) remained statistically significant and stable. This process, however, revealed a critical distinction in the reliability of secondary outcomes. While the benefits for proximal limb control (FMA-SE) withstood adjustments for baseline confounding, the apparent effects on grip strength (GS) and self-reported arm use (MAL) were substantially attenuated and lost statistical significance after meta-regression. Therefore, we can state with confidence that robotic therapy improves core motor and functional outcomes, but its specific efficacy on grip strength remains uncertain and warrants further investigation in more rigorously designed trials.

#### 4.1.13 Impact of baseline heterogeneity on treatment effects

The observed baseline heterogeneity across several outcomes necessitates a careful consideration of the internal validity of our meta-analytic findings. Our meta-regression quantified the substantial confounding effect of these imbalances, which has critical implications for the clinical interpretability of the results. For instance, baseline discrepancies significantly inflated the apparent treatment effect for grip strength. After statistical adjustment, the effect was substantially attenuated and became non-significant (*p* = 0.174). This pattern, consistent across GS subgroups, suggests that unadjusted GS effects are likely overestimates. A similar pattern was observed for MAL-AOU/QOM, where adjustment also nullified significance.

While randomization in the primary RCTs aims to minimize confounding, these analyses indicate that residual imbalances can significantly impact the results for specific outcomes like GS and MAL. For these measures, the stability of the treatment effect is questionable, as statistical significance was contingent upon unadjusted models. This limits the strength of causal inference regarding the robot-assisted therapy’s specific effect on grip strength and self-reported arm use. Clinically, the unadjusted benefits for these outcomes may not be robust and could be attributable, in part, to pre-existing group differences.

In contrast, the treatment effect for FMA-SE demonstrated notable robustness, remaining statistically and clinically significant after adjustment (Hedge’s g = 0.92, 95% CI (0.23, 1.61), *p* = 0.009), which enhances our confidence in its stability and internal validity. Therefore, we recommend that conclusions regarding intervention efficacy be drawn primarily from outcomes, like FMA-SE, that have demonstrated stability against potential baseline confounding. Complete analytical outputs are provided in Supplementary Table S3.

### 4.2 Limitations

Several limitations should be acknowledged: (1) Language bias: The exclusion of non-English publications may restrict the generalizability of findings. (2) Heterogeneity in kinematic assessments: Despite initial inclusion of studies measuring kinematic parameters, methodological inconsistencies (e.g., device variability, lack of standardized metrics) necessitated exclusion of these data, limiting analysis to grip strength outcomes and precluding robust evaluation of motor performance/quality. (3) Sample size constraints: Underpowered subgroup analyses (e.g., acute phase and mild impairment cohorts) may reduce statistical reliability. (4) Baseline imbalances: Significant intergroup differences in baseline scores for select outcomes (e.g., GS, *p* < 0.05) may confound treatment effect interpretations. (5) Uncontrolled spontaneous recovery: Particularly in acute and subacute-phase patients, natural neurological repair processes may conflate therapeutic effects. (6) Cognitive/linguistic confounding: Incomplete reporting of cognitive/communication deficits in included studies risks biasing functional outcome measurements, given the interdependence of motor recovery and higher-order neural functions.

### 4.3 Prospect

Building upon our findings that support a stratified approach to robotic therapy, future research should prioritize the following directions: (1) Differentiating Therapeutic Effects from Natural Recovery through potential implementation of standardized protocols combining Rosenthal’s stability criteria (baseline FMA-UE 5–50 with ≤5-point fluctuation over 14 days) (98); (2) Validation of Patient Selection Biomarkers: Large-scale, prospective trials are needed to validate the proposed stratification criteria (e.g., subacute phase, severe impairment) and to explore integrating multimodal biomarkers, such as neuroimaging (e.g., corticospinal tract integrity via diffusion tensor imaging) and neurophysiological assessments, to create more robust predictive models of treatment response. (3) Advancement of Robotic Technology and Assessment: Efforts should focus on standardizing kinematic outcome measures (e.g., movement smoothness, inter-joint coordination) to capture qualitative motor recovery beyond clinical scales. Furthermore, developing adaptive robotic systems that can personalize therapy in real-time based on patient performance represents a promising frontier. (4) Elucidation of Mechanisms and Long-Term Outcomes: Investigations into the specific neuroplastic mechanisms induced by different robotic modalities are crucial. Finally, studies with extended follow-up periods (≥6–12 months) and robust cost-effectiveness analyses are essential to evaluate the sustained clinical and economic impact of stratified robotic rehabilitation.

## 5 Conclusion

This meta-analysis provides robust evidence that robotic-assisted therapy significantly improves upper limb motor function and activities of daily living after stroke. Crucially, the benefits are not uniform across all patients. A stratified rehabilitation approach is supported by our findings, with patients in the subacute phase and those with severe baseline impairment deriving clinically meaningful improvements. In contrast, the improvements in grip strength and social participation, while statistically significant, are less certain in their clinical importance, partly due to baseline confounding and methodological limitations.

For clinical practice, these results argue for a shift from a one-size-fits-all application to a targeted strategy. We recommend using standardized assessments like the FMA-UE to prioritize subacute and severely impaired stroke survivors for resource-intensive robotic interventions, thereby maximizing functional recovery and healthcare efficiency. The sustainability of such programs can be enhanced through innovative implementation models that address cost barriers.

Future research must now move beyond proving general efficacy. Priorities should include: (1) validating these stratification criteria in prospective, pragmatic trials; (2) elucidating the neuroplastic mechanisms underlying the differential treatment responses observed here, using multimodal neuroimaging; and (3) developing adaptive robotic algorithms that automatically personalize therapy based on real-time performance. Ultimately, these efforts are essential to fully realize the promise of precision neurorehabilitation.

## Data Availability

The original contributions presented in the study are included in the article/Supplementary material, further inquiries can be directed to the corresponding author.
