# Supplementary material for: Effects of robotic-assisted upper extremity therapy for stroke patients in different recovery phases: a systematic review and meta-analysis
Source: Front Neurol. 2026 Jan 12;16:1640522. doi: 10.3389/fneur.2025.1640522 (PMC12832252; doi:10.3389/fneur.2025.1640522)
Supplement: Supplementary file 1 [file Supplementary_file_1.pdf]

SUPPLEMENTARY MATERIALS

TABLE S1 PRISMA 2020 CHECKLIST 3

TABLE S2 ELECTRONIC DATABASE SEARCH STRATEGY 8

FIGURE S1 THE FOREST PLOT OF BASELINE DATA FOR OUTCOME MEASURES 14

FIGURE S1-1 THE FOREST PLOT OF BASELINE DATA FOR PRIMARY OUTCOMES 14
FIGURE S1-2 THE FOREST PLOT OF BASELINE DATA FOR PRIMARY OUTCOMES (GS) 14
FIGURE S1-3 THE FOREST PLOT OF BASELINE DATA FOR SECONDARY OUTCOMES (FMA-SE, FMA-WH, FMA-H) 15
FIGURE S1-4 THE FOREST PLOT OF BASELINE DATA FOR MAL (MAL-AOU, MAL-QOM) 15

FIGURE S2 THE FOREST PLOT OF ENDPOINT AND FOLLOW-UP DATA FOR OUTCOME MEASURES 16

FIGURE S2-1 THE FOREST PLOT OF ENDPOINT DATA FOR PRIMARY OUTCOMES 16
FIGURE S2-2 THE FOREST PLOT OF ENDPOINT DATA FOR PRIMARY OUTCOMES (GS) 16
FIGURE S2-3 THE FOREST PLOT OF ENDPOINT DATA FOR SECONDARY OUTCOMES (FMA-SE, FMA-WH, FMA-H) 17
FIGURE S2-4 THE FOREST PLOT OF ENDPOINT DATA FOR SECONDARY OUTCOMES (MAL-AOU, MAL-QOM) 17
FIGURE S2-5 THE FOREST PLOT OF FOLLOW-UP DATA FOR FMA-UE 18

TABLE S3 COMPARATIVE ANALYSIS OF BASELINE DISCREPANCIES ACROSS OUTCOMES IN PROCESSED DATASETS 19

TABLE S3-1 META-REGRESSION AND POOLED EFFECT SIZE ANALYSIS OF BASELINE COVARIATES ON OUTCOMES 19
TABLE S3-2 IDENTIFICATION OF SOURCES CONTRIBUTING TO BASELINE IMBALANCE IN OUTCOMES 19
TABLE S3-3 LEAVE-ONE-OUT SENSITIVITY ANALYSIS (GS BASELINE) 20
TABLE S3-4 LEAVE-ONE-OUT SENSITIVITY ANALYSIS (GS -CHRONIC BASELINE) 20
TABLE S3-5 LEAVE-ONE-OUT SENSITIVITY ANALYSIS (GS AGE≤ 60 YEARS BASELINE) 21
TABLE S3-6 LEAVE-ONE-OUT SENSITIVITY ANALYSIS (GS DURATION LENGTH<24 SESSIONS BASELINE) 21
TABLE S3-7 LEAVE-ONE-OUT SENSITIVITY ANALYSIS (MBI SESSION LENGTH≤30 MIN BASELINE) 22
TABLE S3-8 RESULTS OF META REGRESSION ANALYSIS (FMA-SE) 22
TABLE S3-9 LEAVE-ONE-OUT SENSITIVITY ANALYSIS (FMA-SE BASELINE) 22
TABLE S3-10 RESULTS OF META REGRESSION ANALYSIS (MAL-AOU) 23
TABLE S3-11 LEAVE-ONE-OUT SENSITIVITY ANALYSIS (MAL-AOU BASELINE) 23
TABLE S3-12 RESULTS OF META REGRESSION ANALYSIS (MAL-QOM) 24
TABLE S3-13 LEAVE-ONE-OUT SENSITIVITY ANALYSIS (MAL-QOM BASELINE) 24

TABLE S4 PUBLICATION BIAS RESULTS 26

FIGURE S3 TRIM AND FILL METHOD AND FUNNEL PLOT 27

FIGURE S3-1 FUNNEL PLOT OF FMA-UE (CHRONIC) AFTER TRIM AND FILL METHOD 27
FIGURE S3-2 FUNNEL PLOT OF FMA-UE (AGE≤60 YEARS) AFTER TRIM AND FILL METHOD 27
FIGURE S3-3 FUNNEL PLOT OF GS AFTER TRIM AND FILL METHOD 28

Figure S3-4 Funnel plot of GS (different phases) after trim and fill method........................ 29
Figure S3-5 Funnel plot of GS (age≤60 years) after trim and fill method 29
TABLE S5 DETAILED ORIGINAL AND RECLASSIFIED PHASES FOR ALL STUDIES 31
TABLE S6 DETAILED RESULTS OF SENSITIVITY ANALYSIS POST HIGH-RISK STUDY EXCLUSION 34
TABLE S7 ABBREVIATIONS 36

Table S1 PRISMA 2020 checklist

Section and Topic	Item #	Checklist item	Location where item is reported
**TITLE**			
Title	1	Identify the report as a systematic review.	Title
**ABSTRACT**			
Abstract	2	See the PRISMA 2020 for Abstracts checklist.	Abstract
**INTRODUCTION**			
Rationale	3	Describe the rationale for the review in the context of existing knowledge.	Introduction
Objectives	4	Provide an explicit statement of the objective(s) or question(s) the review addresses.	Introduction
**METHODS**			
Eligibility criteria	5	Specify the inclusion and exclusion criteria for the review and how studies were grouped for the syntheses.	Materials and Methods
Information sources	6	Specify all databases, registers, websites, organisations, reference lists and other sources searched or consulted to identify studies. Specify the date when each source was last searched or consulted.	Materials and Methods
Search strategy	7	Present the full search strategies for all databases, registers and websites, including any filters and limits used.	Materials and Methods
Selection process	8	Specify the methods used to decide whether a study met the inclusion criteria of the review, including how many reviewers screened each record and each report retrieved, whether they worked independently, and if applicable, details of automation tools used in the process.	Materials and Methods
Data collection process	9	Specify the methods used to collect data from reports, including how many reviewers collected data from each report, whether they worked	Materials and Methods

Section and Topic	Item #	Checklist item	Location where item is reported
		independently, any processes for obtaining or confirming data from study investigators, and if applicable, details of automation tools used in the process.	
Data items	10a	List and define all outcomes for which data were sought. Specify whether all results that were compatible with each outcome domain in each study were sought (e.g. for all measures, time points, analyses), and if not, the methods used to decide which results to collect.	Materials and Methods
	10b	List and define all other variables for which data were sought (e.g. participant and intervention characteristics, funding sources). Describe any assumptions made about any missing or unclear information.	Materials and Methods
Study risk of bias assessment	11	Specify the methods used to assess risk of bias in the included studies, including details of the tool(s) used, how many reviewers assessed each study and whether they worked independently, and if applicable, details of automation tools used in the process.	Materials and Methods
Effect measures	12	Specify for each outcome the effect measure(s) (e.g. risk ratio, mean difference) used in the synthesis or presentation of results.	Materials and Methods
Synthesis methods	13a	Describe the processes used to decide which studies were eligible for each synthesis (e.g. tabulating the study intervention characteristics and comparing against the planned groups for each synthesis (item #5)).	Materials and Methods
	13b	Describe any methods required to prepare the data for presentation or	Materials and Methods

Section and Topic	Item #	Checklist item	Location where item is reported
		synthesis, such as handling of missing summary statistics, or data conversions.	
	13c	Describe any methods used to tabulate or visually display results of individual studies and syntheses.	Materials and Methods
	13d	Describe any methods used to synthesize results and provide a rationale for the choice(s). If meta-analysis was performed, describe the model(s), method(s) to identify the presence and extent of statistical heterogeneity, and software package(s) used.	Materials and Methods
	13e	Describe any methods used to explore possible causes of heterogeneity among study results (e.g. subgroup analysis, meta-regression).	Materials and Methods
	13f	Describe any sensitivity analyses conducted to assess robustness of the synthesized results.	Materials and Methods
Reporting bias assessment	14	Describe any methods used to assess risk of bias due to missing results in a synthesis (arising from reporting biases).	Materials and Methods
Certainty assessment	15	Describe any methods used to assess certainty (or confidence) in the body of evidence for an outcome.	Materials and Methods
**RESULTS**			
Study selection	16a	Describe the results of the search and selection process, from the number of records identified in the search to the number of studies included in the review, ideally using a flow diagram.	Results
	16b	Cite studies that might appear to meet the inclusion criteria, but which were excluded, and explain why they were excluded.	Results

Section and Topic	Item #	Checklist item	Location where item is reported
Study characteristics	17	Cite each included study and present its characteristics.	Results
Risk of bias in studies	18	Present assessments of risk of bias for each included study.	Results
Results of individual studies	19	For all outcomes, present, for each study: (a) summary statistics for each group (where appropriate) and (b) an effect estimate and its precision (e.g. confidence/credible interval), ideally using structured tables or plots.	Results
Results of syntheses	20a	For each synthesis, briefly summarise the characteristics and risk of bias among contributing studies.	Results
	20b	Present results of all statistical syntheses conducted. If meta-analysis was done, present for each the summary estimate and its precision (e.g. confidence/credible interval) and measures of statistical heterogeneity. If comparing groups, describe the direction of the effect.	Results
	20c	Present results of all investigations of possible causes of heterogeneity among study results.	Results
	20d	Present results of all sensitivity analyses conducted to assess the robustness of the synthesized results.	Results, Supplementary Material
Reporting biases	21	Present assessments of risk of bias due to missing results (arising from reporting biases) for each synthesis assessed.	Results, Supplementary Material
Certainty of evidence	22	Present assessments of certainty (or confidence) in the body of evidence for each outcome assessed.	Results, Supplementary Material
**DISCUSSION**			
Discussion	23a	Provide a general interpretation of the results in the context of other evidence.	Discussion

Section and Topic	Item #	Checklist item	Location where item is reported
	23b	Discuss any limitations of the evidence included in the review.	Discussion
	23c	Discuss any limitations of the review processes used.	Discussion
	23d	Discuss implications of the results for practice, policy, and future research.	Discussion
**OTHER INFORMATION**			
Registration and protocol	24a	Provide registration information for the review, including register name and registration number, or state that the review was not registered.	Materials and Methods
	24b	Indicate where the review protocol can be accessed, or state that a protocol was not prepared.	Materials and Methods
	24c	Describe and explain any amendments to information provided at registration or in the protocol.	Materials and Methods
Support	25	Describe sources of financial or non-financial support for the review, and the role of the funders or sponsors in the review.	Funding
Competing interests	26	Declare any competing interests of review authors.	Conflict of Interest
Availability of data, code and other materials	27	Report which of the following are publicly available and where they can be found: template data collection forms; data extracted from included studies; data used for all analyses; analytic code; any other materials used in the review.	Supplementary Material

Table S2 Electronic database search strategy

Database	Search strategy		Deadline	retrieval results
PubMed	#1	"stroke"[MeSH Terms] OR "acute stroke"[Title/Abstract] OR "subacute stroke"[Title/Abstract] OR "subacute stroke"[Title/Abstract] OR "chronic stroke"[Title/Abstract] OR "infarction"[Title/Abstract] OR "intracranial hemorrhage"[Title/Abstract] OR "hemiplegia"[Title/Abstract] OR "cerebral vascular accident"[Title/Abstract] OR "cerebrovascular accident"[Title/Abstract] OR "cerebrovascular apoplexy"[Title/Abstract] OR "brain vascular accident"[Title/Abstract] OR "Apoplexy"[Title/Abstract]	02/09/2024	286 studies
	#2	"upper extremity"[MeSH Terms] OR "upper-limb"[Title/Abstract] OR "arm"[Title/Abstract] OR "forearm"[Title/Abstract] OR "shoulder"[Title/Abstract] OR "elbow"[Title/Abstract] OR "wrist"[Title/Abstract] OR "hand"[Title/Abstract]		
	#3	"robotics"[MeSH Terms] OR "rehabilitation robotics"[Title/Abstract] OR "robotic*"[Title/Abstract] OR "robot*"[Title/Abstract] OR "robot-mediated"[Title/Abstract] OR "robot-supported"[Title/Abstract] OR "endeffector"[Title/Abstract] OR "exoskeleton"[Title/Abstract] OR "therapy computer assisted"[Title/Abstract]		

	**#4**	"clinical trial"[Publication Type] OR "randomized controlled trials"[Title/Abstract] OR "randomized controlled study"[Title/Abstract] OR "RCT"[Title/Abstract] OR "clinical trial"[Title/Abstract]		
	**Search algorithm**	#1 AND #2 AND #3 AND #4		
**Embase**	**#1**	stroke'/exp OR 'stroke' OR 'acute stroke'/exp OR 'acute stroke' OR 'subacute stroke'/exp OR 'subacute stroke' OR 'sub-acute stroke' OR 'chronic stroke'/exp OR 'chronic stroke' OR 'infarction'/exp OR 'infarction' OR 'intracranial hemorrhage'/exp OR 'intracranial hemorrhage' OR 'hemiplegia'/exp OR 'hemiplegia' OR 'cerebral vascular accident'/exp OR 'cerebral vascular accident' OR 'cerebrovascular accident'/exp OR 'cerebrovascular accident' OR 'cerebrovascular apoplexy' OR 'brain vascular accident'/exp OR 'brain vascular accident' OR 'apoplexy'/exp OR 'apoplexy'	02/09/2024	793 studies
	**#2**	upper extremity'/exp OR 'upper extremity' OR 'upper-limb'/exp OR 'upper-limb' OR 'arm'/exp OR 'arm' OR 'forearm'/exp OR 'forearm' OR 'shoulder'/exp OR 'shoulder' OR 'elbow'/exp OR 'elbow' OR 'wrist'/exp OR 'wrist' OR 'hand'/exp OR 'hand'		
	**#3**	robotics'/exp OR 'robotics' OR 'rehabilitation robotics' OR 'robotic*' OR 'robot*' OR 'robot-mediated' OR 'robot-supported' OR 'endeffector' OR 'exoskeleton'/exp OR 'exoskeleton' OR 'therapy		

		computer-assisted'/exp OR 'therapy computer-assisted'		
	#4	randomized controlled trials'/exp OR 'randomized controlled trials' OR 'randomized controlled study'/exp OR 'randomized controlled study' OR 'rct' OR 'clinical trial'/exp OR 'clinical trial'		
	**Search algorithm**	#1 AND #2 AND #3 AND #4		
**Cochrane Library**	#1	(stroke):ti,ab,kw OR (subacute stroke):ti,ab,kw OR (acute stroke):ti,ab,kw OR (chronic stroke):ti,ab,kw OR (sub-acute stroke):ti,ab,kw OR (infarction):ti,ab,kw OR (intracranial hemorrhage):ti,ab,kw OR (hemiplegia):ti,ab,kw OR (cerebral vascular accident):ti,ab,kw OR (Cerebrovascular Accident):ti,ab,kw OR (Cerebrovascular Apoplexy):ti,ab,kw OR (Brain Vascular Accident):ti,ab,kw OR (Apoplexy):ti,ab,kw	02/09/2024	663 studies
	#2	(upper extremity):ti,ab,kw OR (upper-limb):ti,ab,kw OR (arm) :ti,ab,kw OR (forearm):ti,ab,kw OR (shoulder):ti,ab,kw OR (elbow):ti,ab,kw OR (wrist):ti,ab,kw OR (hand):ti,ab,kw		
	#3	(robotics):ti,ab,kw OR (Rehabilitation robotics):ti,ab,kw OR (robotic*):ti,ab,kw OR (robot*):ti,ab,kw OR (robot-		

		mediated):ti,ab,kw OR (robot-supported):ti,ab,kw OR (endeffector):ti,ab,kw OR (exoskeleton):ti,ab,kw OR (therapy computer-assisted):ti,ab,kw		
	**#4**	(randomized controlled trials):ti,ab,kw OR (randomized controlled study):ti,ab,kw OR (RCT):ti,ab,kw OR (clinical trial):ti,ab,kw		
	**Search algorithm**	#1 AND #2 AND #3 AND #4		
**Web of Science**	**#1**	TS=（stroke OR subacute stroke OR acute stroke OR chronic stroke OR sub-acute stroke OR infarction OR intracranial hemorrhage OR hemiplegia OR cerebral vascular accident OR Cerebrovascular Accident OR Cerebrovascular Apoplexy OR Brain Vascular Accident OR Apoplexy） OR TI=(stroke OR subacute stroke OR acute stroke OR chronic stroke OR sub-acute stroke OR infarction OR intracranial hemorrhage OR hemiplegia OR cerebral vascular accident OR Cerebrovascular Accident OR Cerebrovascular Apoplexy OR Brain Vascular Accident OR Apoplexy） OR AB=(stroke OR subacute stroke OR acute stroke OR chronic stroke OR sub-acute stroke OR infarction OR intracranial hemorrhage OR hemiplegia OR cerebral vascular accident OR Cerebrovascular Accident OR Cerebrovascular Apoplexy OR	02/09/2024	326 studies

		Brain Vascular Accident OR Apoplexy)		
	**#2**	TS= (upper extremity OR upper-limb OR arm OR forearm OR shoulder OR elbow OR wrist OR hand) OR TI= (upper extremity OR upper-limb OR arm OR forearm OR shoulder OR elbow OR wrist OR hand) OR AB= (upper extremity OR upper-limb OR arm OR forearm OR shoulder OR elbow OR wrist OR hand)		
	**#3**	TS= (robotics OR Rehabilitation robotics OR robotic* OR robot* OR robot-mediated OR robot-supported OR endeffector OR exoskeleton OR therapy computer-assisted) OR TI= (robotics OR Rehabilitation robotics OR robotic* OR robot* OR robot-mediated OR robot-supported OR endeffector OR exoskeleton OR therapy computer-assisted) OR AB= (robotics OR Rehabilitation robotics OR robotic* OR robot* OR robot-mediated OR robot-supported OR endeffector OR exoskeleton OR therapy computer-assisted)		

	**#4**	TS= (randomized controlled trials OR randomized controlled study OR RCT OR clinical trial) OR TI= (randomized controlled trials OR randomized controlled study OR RCT OR clinical trial) OR AB= (randomized controlled trials OR randomized controlled study OR RCT OR clinical trial)		
	**Search algorithm**	#1 AND #2 AND #3 AND #4		

Figure S1 The forest plot of baseline data for outcome measures

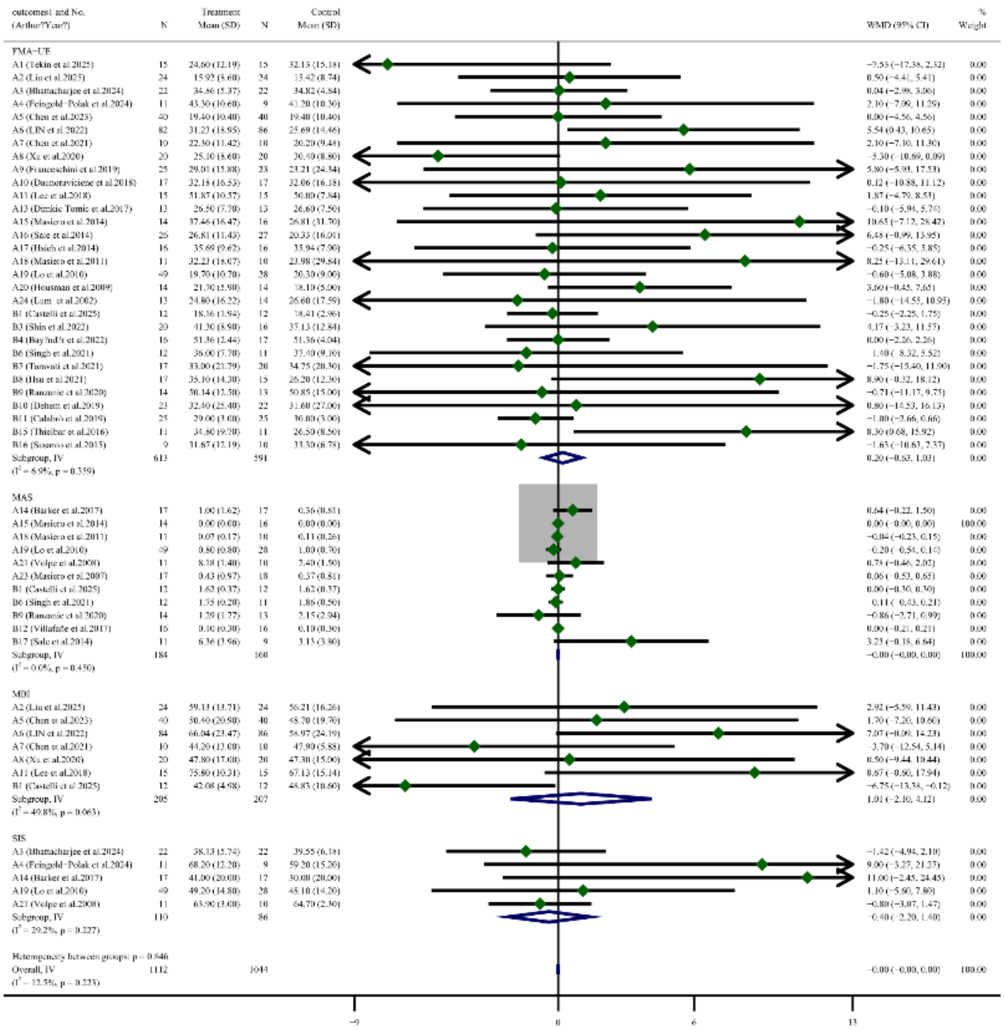

Figure S1-1 The forest plot of baseline data for primary outcomes

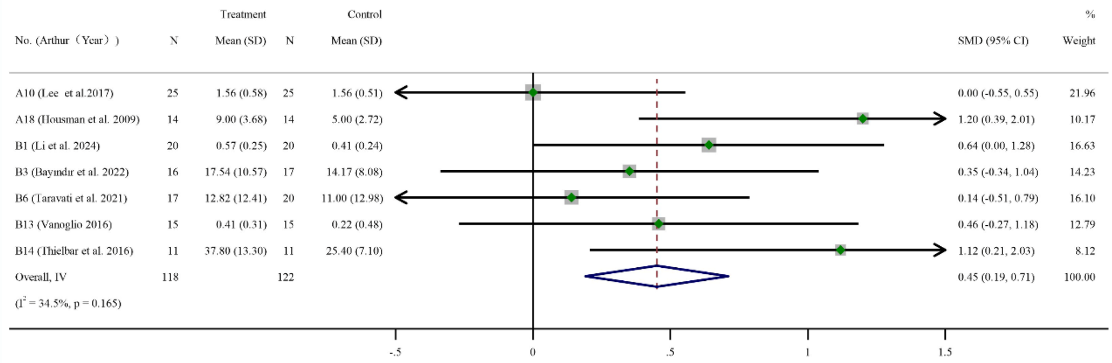

Figure S1-2 The forest plot of baseline data for primary outcomes (GS)

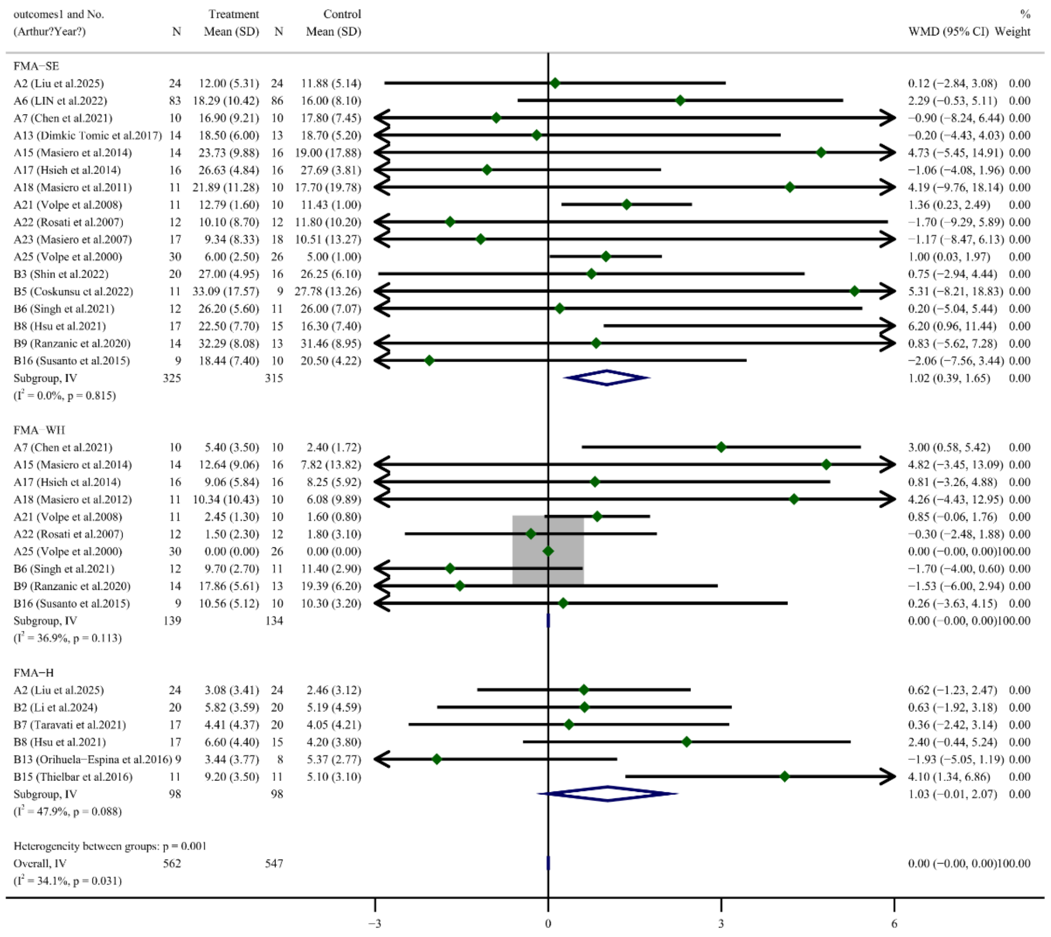

Figure S1-3 The forest plot of baseline data for secondary outcomes (FMA-SE, FMA-WH, FMA-H)

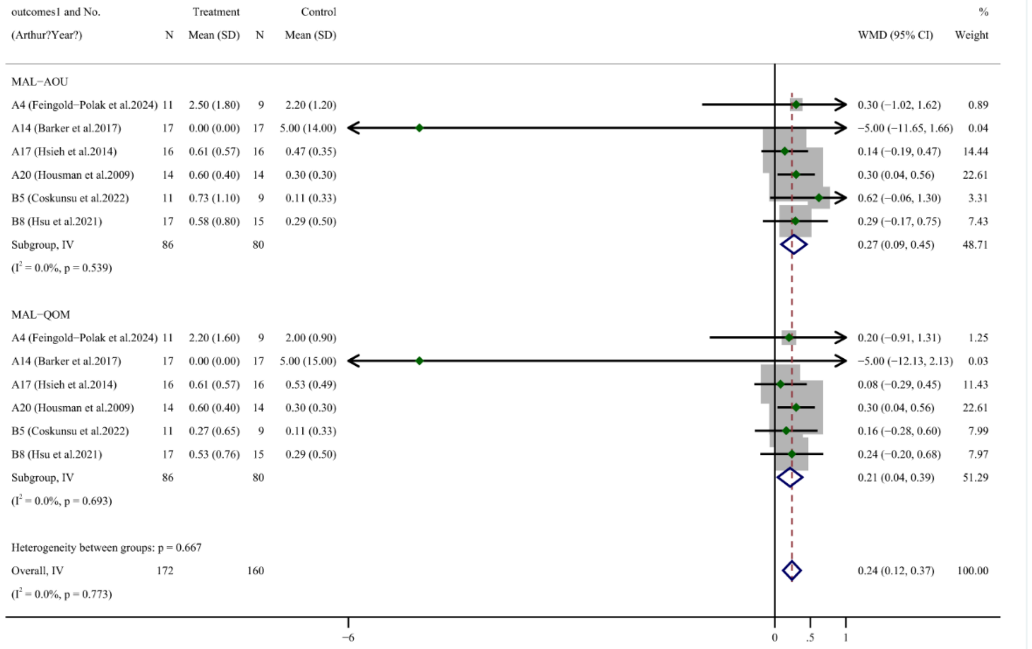

Figure S1-4 The forest plot of baseline data for MAL (MAL-AOU, MAL-QOM)

Figure S2 The forest plot of endpoint and follow-up data for outcome measures

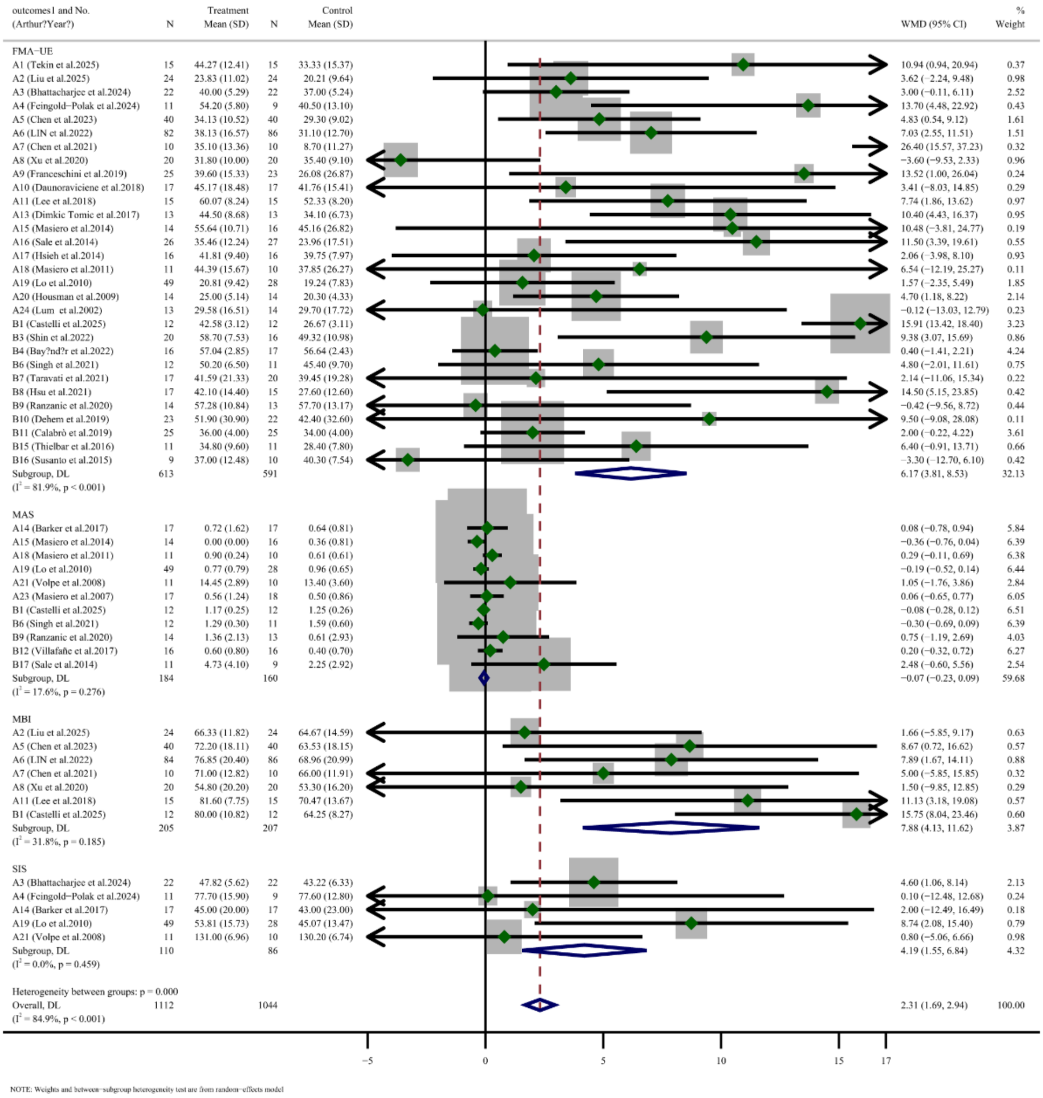

Figure S2-1 The forest plot of endpoint data for primary outcomes

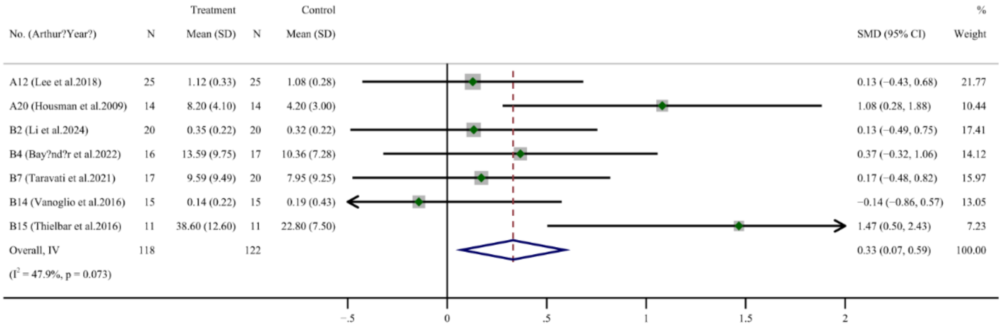

Figure S2-2 The forest plot of endpoint data for primary outcomes (GS)

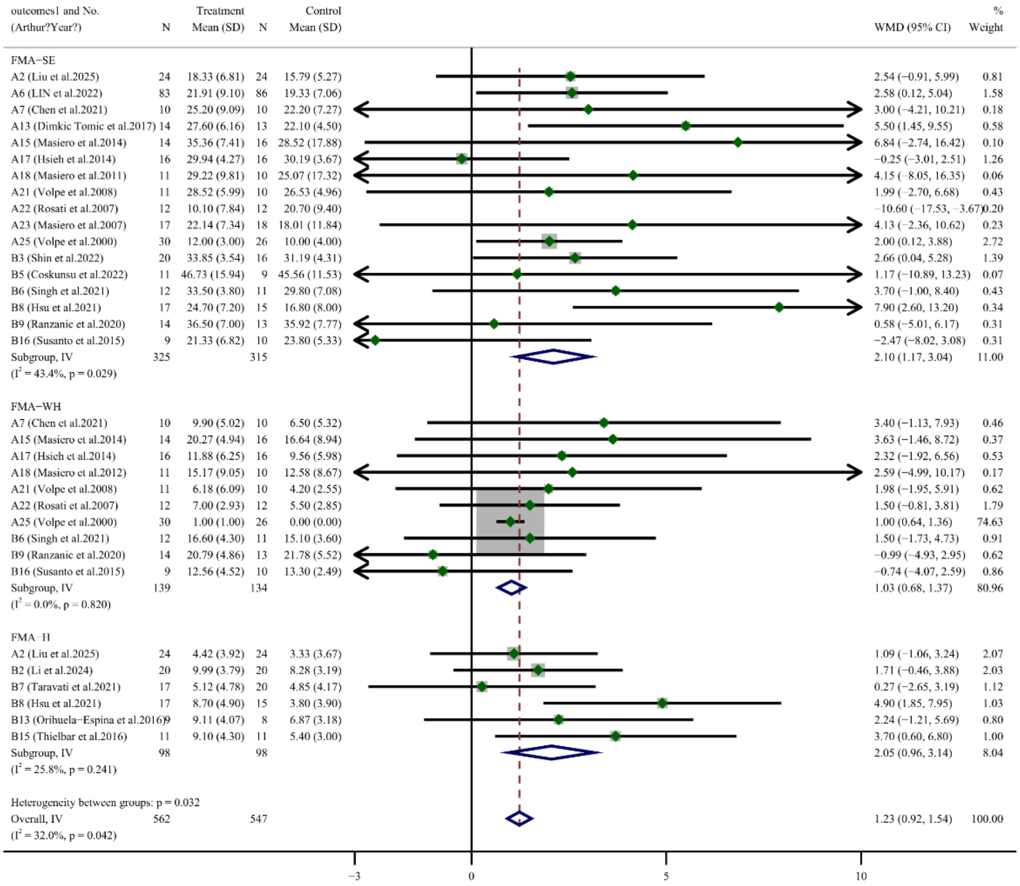

Figure S2-3 The forest plot of endpoint data for secondary outcomes (FMA-SE, FMA-WH, FMA-H)

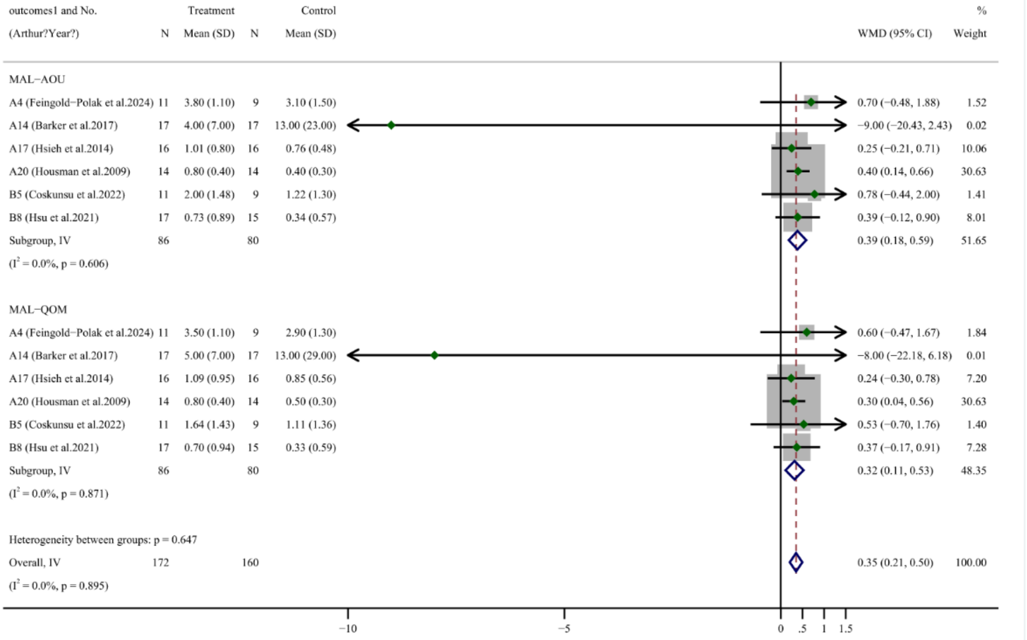

Figure S2-4 The forest plot of endpoint data for secondary outcomes (MAL-AOU,

MAL-QOM)

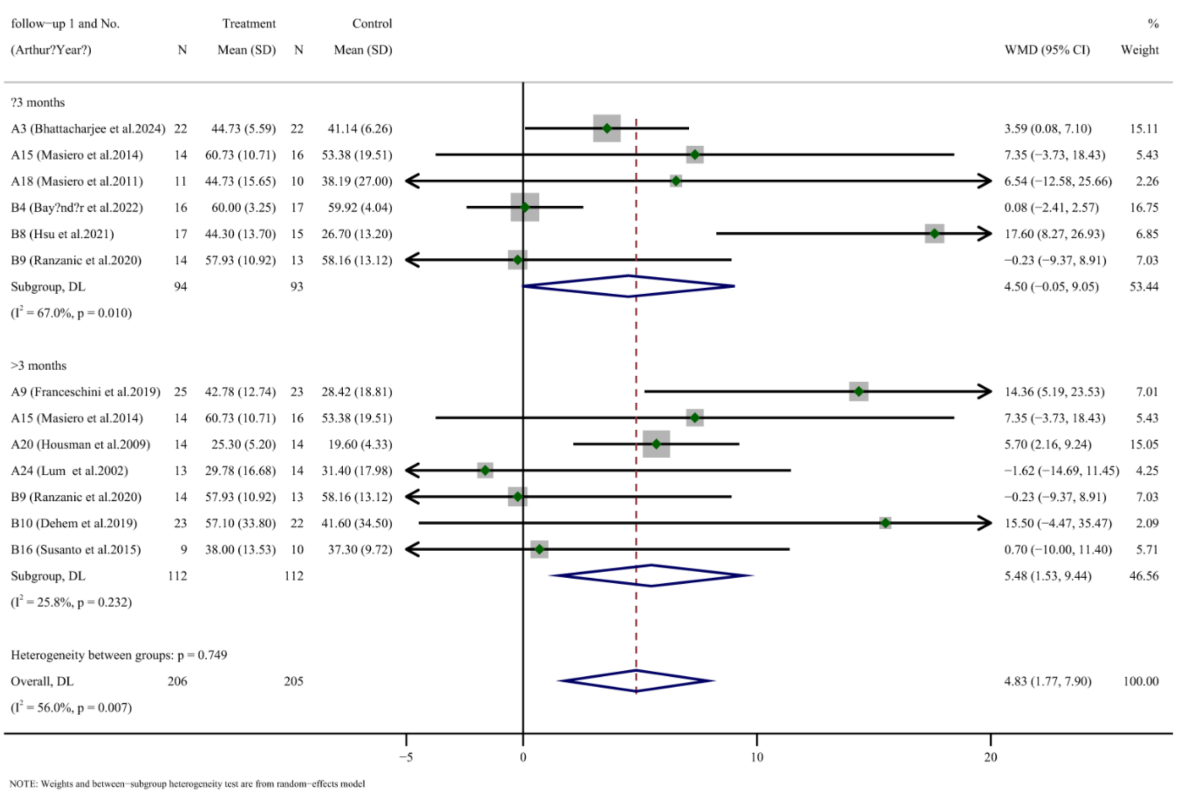

Figure S2-5 The forest plot of follow-up data for FMA-UE

Table S3 Comparative analysis of baseline discrepancies across outcomes in processed datasets

Table S3-1 Meta-Regression and Pooled Effect Size Analysis of Baseline Covariates on Outcomes

**Outcomes**		**Impact of Baseline Imbalance**		**Adjusted Treatment Effect**	
		**Regression coefficient (95% CI)**	***P***	**Pooled Hedge's g (95% CI)**	***P***
**GS**	GS	0.666(0.105,1.227)	0.020	0.224(-0.099,0.548)	0.174
	different phase	0.628(0.093,1.163)	0.022	0.202(-0.093,0.496)	0.179
	chronic	0.799(0.353,1.245)	0.001	-0.014(-0.496,0.469)	0.956
	age≤60 years	0.848(0.398,1.297)	0.001	-0.143(-0.566,0.280)	0.507
	duration length<24 sessions	0.700(0.211,1.190)	0.007	0.086(-0.286,0.458)	0.651
**MBI**	session length≤30 min	1.36(0.03,2.69)	0.046	-1.24(-5.62,3.14)	0.493
**FMA-SE**		0.85(0.65,1.05)	0.000	0.92(0.23,1.61)	0.009
**MAL-AOU**		0.62(0.07,1.17)	0.028	0.21(-0.08,0.50)	0.159
**MAL-QOM**		0.58(0.05,1.11)	0.032	0.19(-0.08,0.46)	0.172

GS: Effect Size: Hedge's g (SMD with small-sample correction). Model: Fixed-effect meta-regression.

MBI, FMA-SE, MAL-AOU, MAL-QOM: Effect Size: Weighted Mean Difference (WMD). Model: Fixed-effect meta-regression ($d_{end}=\beta_0+\beta_1\times d_{base}$)

Table S3-2 Identification of sources contributing to baseline imbalance in outcomes

**Outcome measures**	***P*-value of baseline**	***P*-value of meta regression**	**Sources of baseline discrepancies**
**GS**	0.012	>0.05	Study A20, B4, B7, B15
GS (Subgroup-different phases-chronic)	0.015	>0.05	Study A20, B15

**GS** (Subgroup-age≤60 years)	0.021	>0.05	Study A20, B15
**GS** (Subgroup-duration length<24 sessions)	0.041	>0.05	Study B4, B15
**MBI** (Subgroup-session length≤30 min)	0.019	>0.05	Study A6, A11 and small number of included studies
**FMA-SE**	0.001	>0.05	No obvious sources
**MAL-AOU**	0.001	>0.05	Study A20
**MAL-QOM**	0.016	>0.05	Study A20

Meta-regression REML estimate of between-study variance % residual variation due to heterogeneity. Proportion of between-study variance explained With Knapp-Hartung modification.

Table S3-3 Leave-one-out sensitivity analysis (GS baseline)

**Study Removed**	**Number of studies**	**I^2**	**P-value (Cochran's Q Test)**	**Z-value**	**Pooled SMD (95%CI)**	***P*-value**
**Original Analysis**	7	0.479	0.073	2.505	0.33(0.07,0.59)	0.012
**A12**	6	54.00%	0.054	1.994	0.45(0.01,0.88)	0.046
**A20**	6	35.60%	0.170	1.529	0.27(-0.08,0.62)	0.126
**B2**	6	54.80%	0.050	1.990	0.44(0.01,0.87)	0.047
**B4**	6	56.60%	0.042	1.789	0.40(-0.04,0.83)	0.074
**B7**	6	55.50%	0.047	1.950	0.43(-0.00,0.86)	0.051
**B14**	6	47.90%	0.088	2.323	0.47(0.07,0.86)	0.020
**B15**	6	13.30%	0.329	1.674	0.25(-0.04,0.54)	0.094

The baseline period differences were probably caused by A20, B4, B7 and B15.

Table S3-4 Leave-one-out sensitivity analysis (GS -chronic baseline)

**Study Removed**	**Number of studies**	**I^2**	**P-value (Cochran's Q Test)**	**Z-value**	**Pooled SMD (95%CI)**	***P*-value**

**Original Analysis**	4	0.545	0.086	2.424	0.70(0.13,1.27)	0.015
**A20**	3	60.00%	0.082	1.674	0.59(-0.10,1.28)	0.094
**B4**	3	65.70%	0.054	2.116	0.85(0.06,1.63)	0.034
**B7**	3	47.00%	0.152	2.773	0.91(0.27,1.55)	0.006
**B15**	3	36.20%	0.209	1.900	0.50(-0.02,1.01)	0.057

The baseline period differences were mainly caused by the two studies, A20 and B15.

Table S3-5 Leave-one-out sensitivity analysis (GS age≤ 60 years baseline)

**Study Removed**	**Number of studies**	**I^2**	**P-value (Cochran's Q Test)**	**Z-value**	**Pooled SMD (95%CI)**	***P*-value**
**Original Analysis**	5	0.54	0.069	2.312	0.55(0.08,1.02)	0.021
**A12**	4	54.50%	0.086	2.424	0.70(0.13,1.27)	0.015
**A20**	4	50.50%	0.109	1.714	0.43(-0.06,0.92)	0.087
**B4**	4	65.10%	0.035	2.016	0.63(0.02,1.24)	0.044
**B7**	4	60.80%	0.054	2.269	0.68(0.09,1.26)	0.023
**B15**	4	26.90%	0.251	1.892	0.37(-0.01,0.76)	0.059

The baseline period differences were mainly attributed to the two studies, A20 and B15.

Table S3-6 Leave-one-out sensitivity analysis (GS duration length<24 sessions baseline)

**Study Removed**	**Number of studies**	**I^2**	**P-value (Cochran's Q Test)**	**Z-value**	**Pooled SMD (95%CI)**	***P*-value**
**Original Analysis**	5	0.381	0.167	2.048	0.31(0.01,0.61)	0.041
**A12**	4	49.10%	0.117	2.134	0.38(0.03,0.73)	0.033
**B2**	4	50.50%	0.109	2.101	0.36(0.02,0.70)	0.036
**B4**	4	53.30%	0.093	1.770	0.30(-0.03,0.62)	0.077
**B7**	4	51.90%	0.100	2.036	0.35(0.01,0.68)	0.042
**B15**	4	0.00%	0.953	1.188	0.19(-0.12,0.50)	0.235

The baseline period differences were mainly caused by the two studies, B4 and B15.

Table S3-7 Leave-one-out sensitivity analysis (MBI session length≤30 min baseline)

Study Removed	Number of studies	I^2	P-value (Cochran's Q Test)	Z-value	Pooled WMD (95%CI)	*P*-value
**Original Analysis**	3	0	0.454	2.352	5.91(0.98,10.83)	0.019
**A6**	2	28.00%	0.239	1.408	4.87(-1.91,11.65)	0.159
**A8**	2	0.00%	0.789	2.651	7.67(2.00,13.34)	0.008
**A11**	2	9.50%	0.293	1.627	4.82(-0.99,10.63)	0.104

The baseline period differences were mainly related to the two studies A6 and A11 and the small number of included studies.

Table S3-8 Results of Meta regression analysis (FMA-SE)

	studies	I^2	β	P-value	SMD (95%CI)
**phase**	17	0.00%	0.130	0.708	0.34(-0.59,0.85)
**P&D**	17	0.00%	0.611	0.482	0.85(-1.20,2.42)
**type**	17	0.00%	0.918	0.444	0.91(-2.65,1.22)
**severity**	17	0.00%	0.085	0.914	0.77(-1.58,1.73)
**Session length**	17	0.00%	-0.195	0.821	0.85(-2.00,1.61)
**age**	17	0.00%	0.338	0.684	0.81(-1.40,2.07)
**Duration length**	17	0.00%	-0.092	0.810	0.65(-1.47,1.29)

Meta-regression REML estimate of between-study variance % residual variation due to heterogeneity. Proportion of between-study variance explained With Knapp-Hartung modification.

Table S3-9 Leave-one-out sensitivity analysis (FMA-SE baseline)

Study Removed	Number of studies	I^2	P-value (Cochran's Q Test)	Z-value	Pooled WMD (95%CI)	*P*-value
**Original Analysis**	17	0.00%	0.815	3.161	1.02(0.39,1.65)	0.002
**A2**	16	0.00%	0.784	3.218	1.06(0.41,1.70)	0.001
**A6**	16	0.00%	0.814	2.879	0.95(0.30,1.60)	0.004

**A7**	16	0.00%	0.777	3.194	1.03(0.40,1.66)	0.001
**A13**	16	0.00%	0.781	3.211	1.04(0.41,1.68)	0.001
**A15**	16	0.00%	0.794	3.111	1.00(0.37,1.63)	0.002
**A17**	16	0.00%	0.877	3.379	1.11(0.47,1.75)	0.001
**A18**	16	0.00%	0.773	3.138	1.01(0.38,1.64	0.002
**A21**	16	0.00%	0.794	2.225	0.86(0.10,1.62)	0.026
**A22**	16	0.00%	0.793	3.209	1.04(0.40,1.67)	0.001
**A23**	16	0.00%	0.783	3.200	1.03(0.40,1.66)	0.001
**A25**	16	0.00%	0.759	2.438	1.03(0.20,1.85)	0.015
**B3**	16	0.00%	0.760	3.139	1.02(0.39,1.66)	0.002
**B5**	16	0.00%	0.786	3.129	1.01(0.38,1.64)	0.002
**B6**	16	0.00%	0.766	3.175	1.03(0.39,1.66)	0.001
**B8**	16	0.00%	0.955	2.903	0.94(0.31,1.57)	0.004
**B9**	16	0.00%	0.759	3.152	1.02(0.39,1.65)	0.002
**B16**	16	0.00%	0.839	3.267	1.06(0.42,1.69)	0.001

No obvious source of statistical differences in the baseline period was found.

Table S3-10 Results of Meta regression analysis (MAL-AOU)

	**studies**	**I^2**	**t-value**	**P-value**	**SMD (95%CI)**
phase	6	0.00%	-0.95	0.398	0.18(-0.66,0.32)
P&D	6	0.00%	0.980	0.383	0.20(-0.35,0.74)
type	5	1.78%	-1.00	0.391	0.20(-0.84,0.44)
follow-up	3	58.89%	-0.41	0.751	3.76(-49.38,46.27)
severity	5	0.00%	-0.25	0.818	0.17(-0.59,0.50)
session	6	1.63%	-0.10	0.927	0.26(-0.73,0.68)
age	6	0.00%	1.040	0.357	0.36(-0.63,1.38)
duration	6	0.00%	0.320	0.767	0.18(-0.45,0.56)

Meta-regression REML estimate of between-study variance % residual variation due to heterogeneity. Proportion of between-study variance explained With Knapp-Hartung modification.

Table S3-11 Leave-one-out sensitivity analysis (MAL-AOU baseline)

**Study Removed**	**Number of studies**	**I^2**	**P-value (Cochran's Q Test)**	**Z-value**	**Pooled WMD (95%CI)**	***P*-value**

**Original Analysis**	6	0	0.784	3.218	1.06(0.41,1.70)	0.001
**A4**	5	1.80%	0.396	2.921	0.27(0.09,0.45)	0.003
**A14**	5	0.00%	0.797	2.995	0.27(0.09,0.45)	0.003
**A17**	5	0.00%	0.520	2.979	0.32(0.11,0.54)	0.003
**A20**	5	0.00%	0.409	1.946	0.24(-0.00,0.49)	0.052
**B5**	5	0.00%	0.559	2.581	0.24(0.06,0.43)	0.010
**B8**	5	1.60%	0.397	2.681	0.27(0.07,0.46)	0.007

Study A20 is the main cause of the significant difference in the baseline period.

Table S3-12 Results of Meta regression analysis (MAL-QOM)

	**studies**	**I^2**	**t-value**	**P-value**	**SMD (95%CI)**
**phase**	6	0.00%	0.310	0.770	0.12(-0.30,0.37)
**P&D**	6	0.00%	0.870	0.433	0.21(-0.39,0.75)
**type**	5	0.00%	-0.90	0.435	0.21(-0.87,0.49)
**follow-up**	3	52.82%	-0.37	0.772	3.60(-47.04,44.36)
**severity**	5	0.00%	-0.77	0.498	0.16(-0.65,0.40)
**session**	6	0.00%	-0.12	0.907	0.24(-0.71,0.65)
**age**	6	0.00%	-0.26	0.806	0.24(-0.74,0.61)
**duration**	6	0.00%	0.860	0.439	0.18(-0.34,0.65)

Meta-regression REML estimate of between-study variance % residual variation due to heterogeneity. Proportion of between-study variance explained With Knapp-Hartung modification.

Table S3-13 Leave-one-out sensitivity analysis (MAL-QOM baseline)

**Study Removed**	**Number of studies**	**I^2**	**P-value (Cochran's Q Test)**	**Z-value**	**Pooled WMD (95%CI)**	***P*-value**
**Original Analysis**	6	0	0.693	2.415	0.21(0.04,0.39)	0.016
**A4**	5	0.00%	0.550	2.389	0.22(0.04,0.39)	0.017
**A14**	5	0.00%	0.911	2.449	0.22(0.04,0.39)	0.014
**A17**	5	0.00%	0.664	2.511	0.25(0.06,0.45)	0.012
**A20**	5	0.00%	0.679	1.236	0.15(-0.09,0.38)	0.217
**B5**	5	0.00%	0.561	2.322	0.22(0.04,0.41)	0.020

**B8**	5	0.00%	0.552	2.170	0.21(0.02,0.40)	0.030

Study A20 is the main cause of the significant difference in the baseline period.

Table S4 Publication bias results

Outcomes	Subgroup	Egger's Test P-value	Trim & Fill Adjusted P-value	Interpretation
**FMA-UE**	chronic	0.044	0.000	There is publication bias but the results are robust. Publication bias has no significant impact on the combined results.
	age≤60 years	0.002	0.000	There is publication bias but the results are robust. Publication bias has no significant impact on the combined results.
**GS**	all studies	0.012	0.017	There is publication bias but the results are robust. Publication bias has no significant impact on the combined results.
	different phases	0.005	0.030	There is publication bias but the results are robust. Publication bias has no significant impact on the combined results.
	age≤60 years	0.014	0.013	There is publication bias but the results are robust. Publication bias has no significant impact on the combined results.

Figure S3 Trim and fill method and Funnel plot

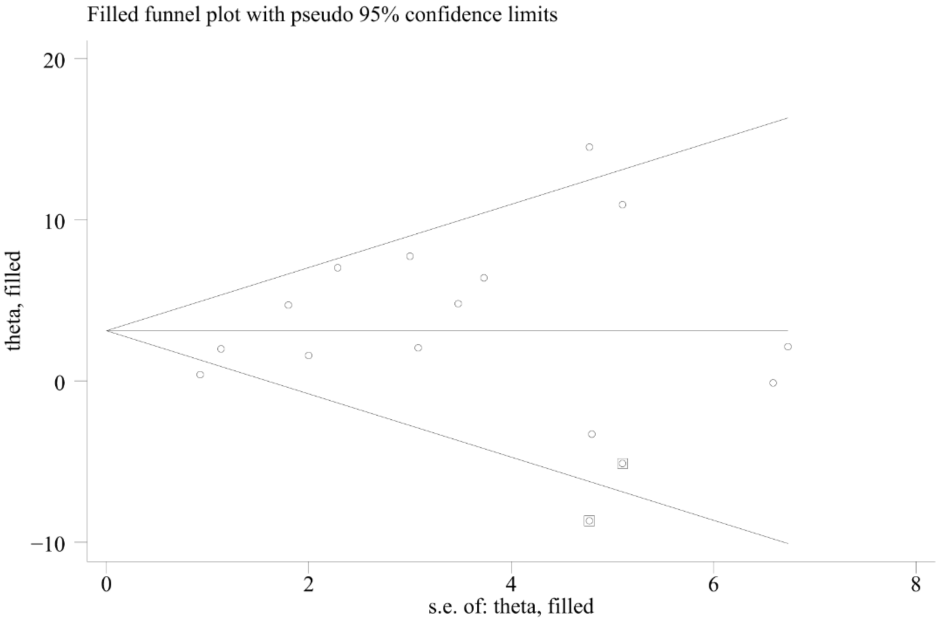

Figure S3-1 Funnel plot of FMA-UE (chronic) after trim and fill method

Trim and fill method-FMAUE: chronic, Egger's test p = 0.044. Using the random effects model, after 4 iterations of the trim and fill method and incorporating 2 additional studies, 95%CI 3.12(1.10,5.14), Q = 33.804, and P = 0.004. Although there was publication bias, the results were robust. Publication bias had no significant impact on the combined results.

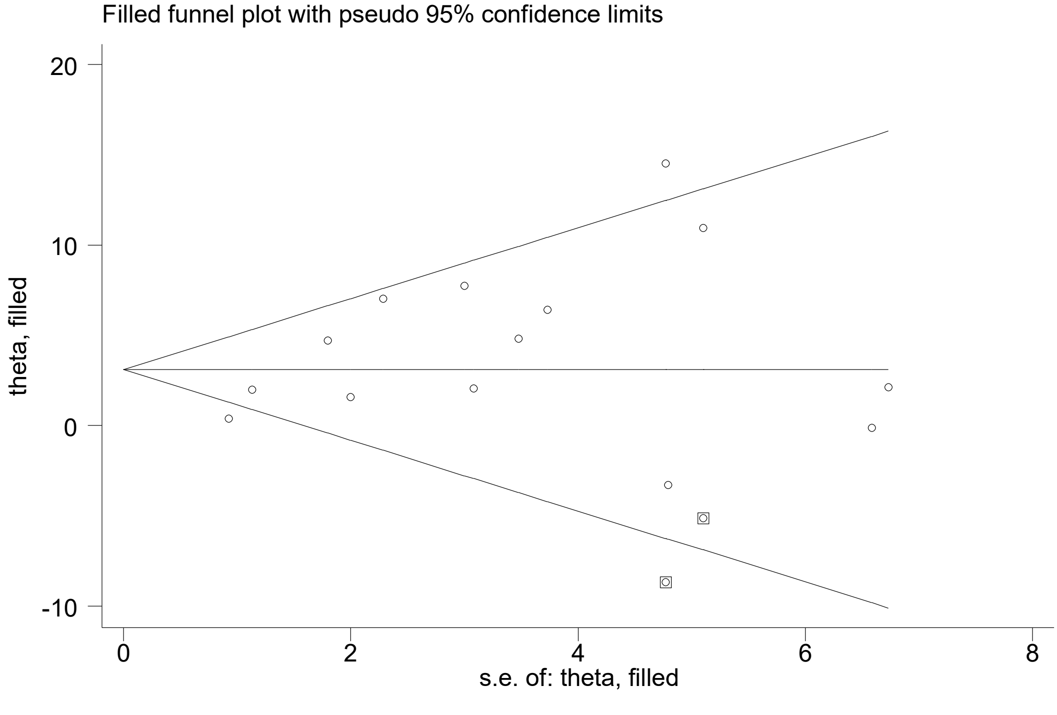

Figure S3-2 Funnel plot of FMA-UE (age≤60 years) after trim and fill method

Trim and fill method-FMA-UE: age ≤ 60 years, Egger's test p = 0.002. After 2 iterations

of the trim and fill method and no adding study, 95%CI=6.30(3.76,8.84), Q=54.975, P=0.000. Although there was publication bias, the results were robust. Publication bias had no significant impact on the combined results.

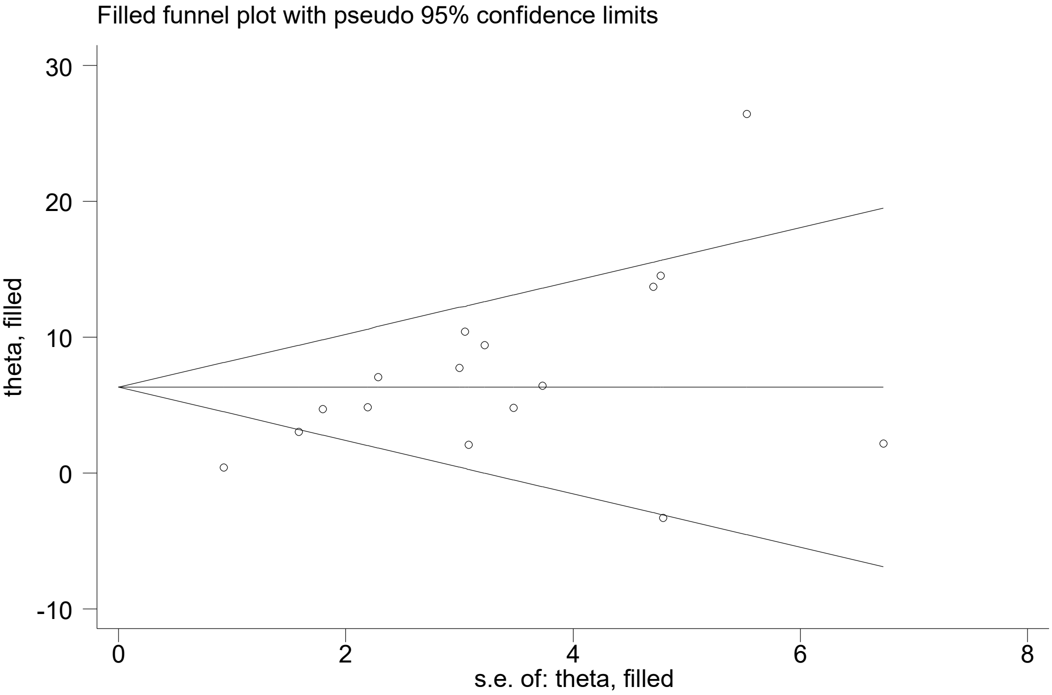

Figure S3-3 Funnel plot of GS after trim and fill method

Trim and fill method-GS, Egger's test p = 0.012. Using the random effects model, after 4 iterations with the trim and fill method and adding 2 studies, 95%CI=0.29 (0.05,0.53), Q=18.569,P=0.017. Although there was publication bias, the results were robust. Publication bias had no significant impact on the combined results.

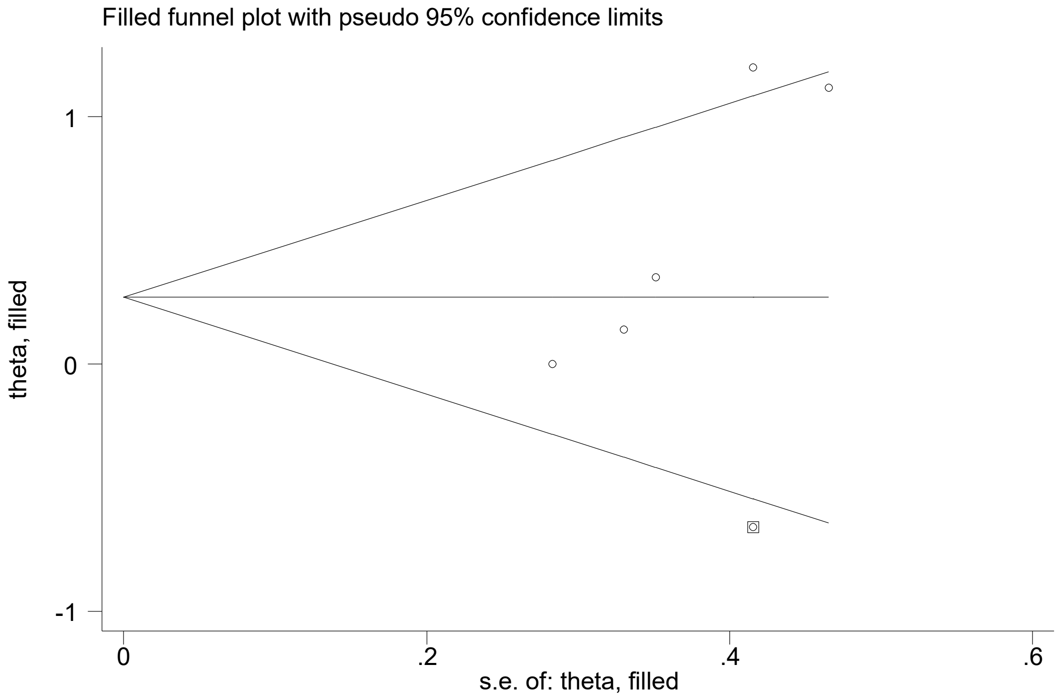

Figure S3-4 Funnel plot of GS (different phases) after trim and fill method

Trim and fill method-GS: different phases, Egger's test p = 0.005. Using a random effects model, after applying the trimming and filling method and conducting 3 iterations,1 additional studies were included. 95%CI=0.30(0.03,0.57)，Q=13.975，P=0.030 Although there was publication bias, the results were robust. Publication bias had no significant impact on the combined results.

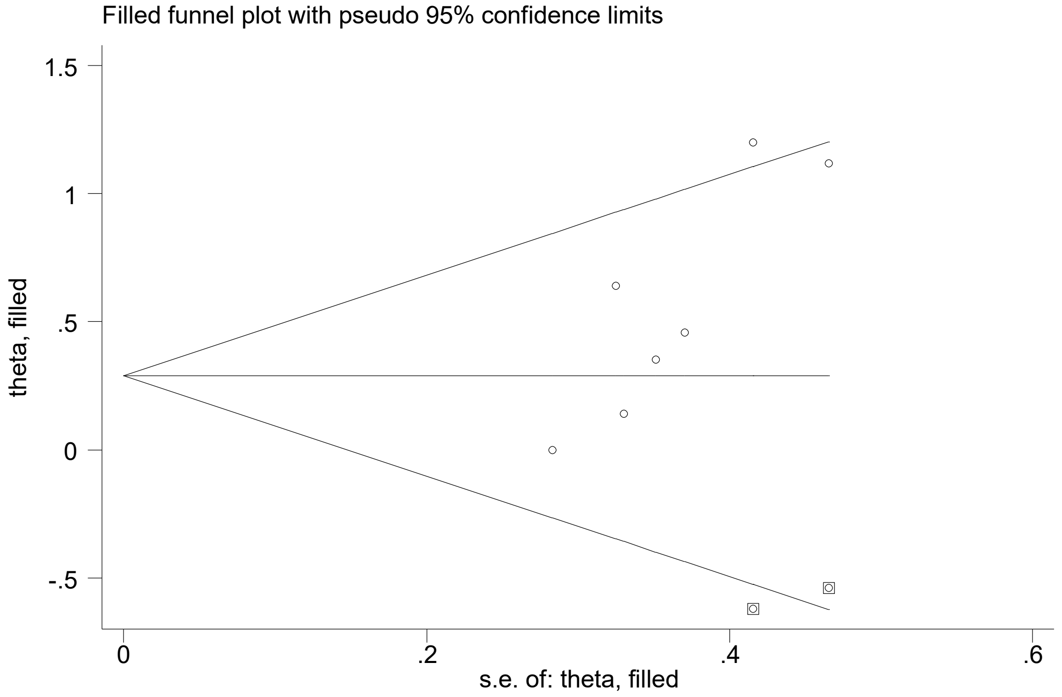

Figure S3-5 Funnel plot of GS (age≤60 years) after trim and fill method

Trim and fill method-GS, for patients aged ≤ 60 years, Egger's test $p = 0.014$. Using the random effects model, after 3 iterations of the trimming and filling method and adding 1 study, 95%CI=0.33(-0.18,0.83)， Q=14.456， P=0.013. Although there is publication bias, the results are robust. Publication bias has no significant impact on the combined results.

Table S5 Detailed original and reclassified phases for all studies

NO.	Arthur (Year)	Sample size			Start of treatment (days) since stroke onset		Phase in the source study	Phase in this study
		Total	EG	CG	EG	CG		
A1	Tekin (2025)	30	15	15	≥182.64	≥182.64	chronic	chronic
A2	Liu (2024)	48	24	24	118.72± 76.1	106.54± 82.19	/	**subacute**
A3	Bhattacharjee et al. 2024	44	22	22	126.02±36.83	124.50±47.79	subacute	subacute
A4	Feingold-Polak et al. 2024	33	11	9	108±56	92±42	/	**subacute**
A5	Chen et al. 2023	80	40	40	50.3±40.3	58.9±35.4	subacute	subacute
A6	LIN et al. 2022	168	82	86	142.30±162.84	158.23±178.20	/	**chronic**
A7	Chen et al. 2021	20	10	10	74.90±54.52	50.10±38.24	subacute	subacute
A8	Xu et al.2020	40	20	20	51.0 ± 19.1	47.2 ± 24.0	subacute	subacute
A9	Franceschini et al. 2019	48	25	23	30.64±3.92	31±3.17	subacute	subacute
A10	Daunoraviciene et al.2018	34	17	17	60.48 ± 24.71	67.55 ± 47.46	/	**subacute**
A11	Lee et al.2018	30	15	15	>213.08	>213.08	/	**chronic**
A12	Lee et al.2017	50	25	25	15.40±8.05	14.40±6.95	/	**acute**
A13	Dimkic Tomic et al. 2017	26	13	13	35.3 ± 9.7	37.3 ± 7.7	subacute	subacute
A14	Barker et al. 2017	50	17	17	43.9±21.7	34.7±31.2	subacute	subacute
A15	Masiero et al. 2014	30	14	16	8.34 ±3.2	10.23±2.4	acute	acute
A16	Sale et al.2014	53	26	27	30±7	30±7	subacute	subacute
A17	Hsieh et al. 2014	48	16	16	717.17±469.69	846.54±580.49	/	**chronic**

A18	Masiero et al. 2011	21	11	10	10.1±4.5	12.5±5.2	acute	acute
A19	Lo et al.2010	127	49	28	1281.6±1424	2207.2±1780	/	**chronic**
A20	Housman et al. 2009	28	14	14	2572.18 ±2931.37	3421.46 ±3911.54	chronic	chronic
A21	Volpe et al. 2008	21	11	10	1065.4 ± 213.08	1217.6 ± 334.84	chronic	chronic
A22	Rosati et al. 2007	24	12	12	5.1±2.1	5.5±3.2	subacute	**acute**
A23	Masiero et al. 2007	35	17	18	<7	<7	acute	acute
A24	Lum et al.2002	27	13	14	919.29±188.73	876.67±191.78	chronic	chronic
A25	Volpe et al. 2000	56	30	26	22.5±1.3	26.0±1.4	/	**acute**
B1	Castelli (2025)	24	12	12	133.33 ± 49.92	136.98± 57.53	/	**subacute**
B2	Li et al. 2024	40	20	20	70.55 ± 49.37	67.35 ± 47.31	/	**subacute**
B3	Shin et al. 2022	36	20	16	24.70 ±16.26	34.00 ±25.49	subacute	subacute
B4	Bayındır et al. 2022	33	16	17	494.46±791.75	242.80±270.68	subacute	**chronic**
B5	Coskunsu 2022	20	11	9	<28	<28	acute	acute
B6	Singh et al. 2021	23	12	11	420.07± 277.00	313.53 ±152.20	/	**chronic**
B7	Taravati et al. 2021	37	17	20	333.01 ± 244.13	385.07± 256.31	/	**chronic**
B8	Hsu et al. 2021	32	17	15	718.38± 484.00	1104.97± 897.98	chronic	chronic
B9	Ranzanic 2020	27	14	13	21.98±10.57	21.56±9.24	subacute	**acute**
B10	Dehem 2019	45	23	22	28.1 ±4.4	27.5±6.6	acute	acute
B11	Calabrò et al. 2019	50	25	25	182.4	182.4	chronic	chronic
B12	Villafañe et al. 2017	32	16	16	<182.64	<182.64	acute	**subacute**

B13	Orihuela-Espina et al. 2016	17	9	8	74.27 ±26.79	66.36±38.05	subacute	subacute
B14	Vanoglio 2016	30	15	15	15.2 ± 6.8	17.8 ± 7.9	subacute	**acute**
B15	Thielbar et al. 2016	22	11	11	2891.8±3470.16	1461.12±1430.68	subacute	**chronic**
B16	Susanto et al. 2015	19	9	10	499.22±176.55	490.08±155.24	chronic	chronic
B17	Sale 2014	20	11	9	30 ± 7	30 ± 7	acute	acute

Table S6 Detailed results of sensitivity analysis post high-Risk study exclusion

**Outcome**	**Studies**	**Participants**	**baseline**				**end of treatment**			
			**Heterogeneity**		**WMD (95%CI)**	***P*-value**	**Heterogeneity**		**WMD (95%CI)**	***P*-value**
			**I^2**	***P*-value** (Cochran's Q)			**I^2**	***P*-value** (Cochran's Q)		
**FMA-UE**	30	1204	6.90%	0.359	0.20(-0.63,1.03)	0.640	81.90%	0.000	6.17(3.81,8.53)	0.000
**FMA-UE'**	28	1137	13.20%	0.267	0.23(-0.67,1.13)	0.615	79.20%	0.000	6.55(4.09,9.01)	0.000
**MAS**	11	344	0.00%	0.450	-0.00(-0.00,0.00)	1.000	17.60%	0.276	-0.08(-0.21,0.05)	0.245
**MAS'**	10	310	0.00%	0.058	-0.00(-0.00,0.00)	1.000	25.00%	0.213	-0.07(-0.24,0.11)	0.447
**GS**	7	240	47.90%	0.073	0.33(0.07,0.59) *	***0.012***	34.50%	0.165	0.45(0.19,0.71) *	0.001
**GS'**	5	157	63.20%	0.028	0.48(-0.07,1.02) *	0.085	26.00%	0.248	0.63(0.30,0.95) *	0.000
**MBI**	7	412	49.80%	0.063	1.01(-2.10,4.12)	0.524	31.80%	0.185	8.00(4.96,11.03)	0.000
**MBI'**	7	412	49.80%	0.063	1.01(-2.10,4.12)	0.524	31.80%	0.185	7.88(4.13,11.62)	0.000
**SIS**	5	196	29.20%	0.227	0.13(-2.55,2.80)	0.925	0.00%	0.459	4.19(1.55,6.84)	0.002
**SIS'**	4	162	0.00%	0.418	-0.61(-2.43,1.21)	0.511	15.10%	0.316	4.22(1.92,7.34)	0.008
**FMA-SE**	17	640	0.00%	0.815	1.02(0.39,1.65)	***0.002***	43.40%	0.029	2.10(1.17,3.04)	0.000
**FMA-SE'**	17	640	0.00%	0.815	1.02(0.39,1.65)	***0.002***	43.40%	0.029	2.10(1.17,3.04)	0.000
**FMA-WH**	10	273	36.90%	0.113	0.00(-0.00,0,00)	1.000	0.00%	0.820	1.03(0.69,1.37)	0.000
**FMA-WH'**	10	273	36.90%	0.113	0.00(-0.00,0,00)	1.000	0.00%	0.820	1.03(0.69,1.37)	0.000
**FMA-H**	6	196	47.90%	0.088	1.03(-0.01,2.07)	0.051	25.80%	0.241	2.06(0.97,3.15)	0.000
**FMA-H'**	5	179	30.00%	0.221	1,40(0.30,2.49)	0.013	40.60%	0.151	2.03(0.89,3.18)	0.001
**MAL-AOU**	6	166	0.00%	0.539	0.27(0.09,0.45)	***0.003***	0.00%	0.606	0.39(0.18,0.59)	0.000

**MAL-AOU'**	5	132	0.00%	0.797	0.27(0.09,0.45)	***0.003***	0.00%	0.906	0.39(0.19,0.59)	0.000
**MAL-QOM**	6	166	0.00%	0.693	0.21(0.04,0.39)	***0.016***	0.00%	0.871	0.32(0.11,0.53)	0.003
**MAL-QOM'**	5	132	0.00%	0.911	0.22(0.04,0.39)	0.014	0.00%	0.972	0.32(0.11,0.53)	0.003

Baseline P-values in bold italics indicate significant between-group differences (P<0.05).

GS outcome (marked with *) used SMD due to methodological heterogeneity in measurement.

Table S7 Abbreviations

Abbreviations	
FMA-UE	Fugl-Meyer Upper Extremity Motor Assessment
MAS	Modified Ashworth Scale
GS	grip strength
MBI	modified Barthel Index
SIS	Stroke Impact Scale
FMA-SE	Fugl-Meyer Upper Extremity Motor Assessment-proximal (shoulder, elbow & forearm)
FMA-WH	Fugl-Meyer Upper Extremity Motor Assessment-distal (wrist & hand)
FMA-H	Fugl-Meyer Upper Extremity Motor Assessment-hand
MAL-AOU	Motor Activity Log -amount of use
MAL-QOM	Motor Activity Log -quality of movement
EXO	exoskeleton robotics
EE	end-effector robotics
SRG	soft robotic gloves
EG	Experimental Group
CG	Conventional Group
MCID	Minimum Clinically Important Difference
rTMS	repetitive transcranial magnetic stimulation
VR	virtual reality
BCI	brain-computer interfaces
RT	robotic-assisted upper extremity therapy
CT	conventional therapy
RCT	randomized controlled trial
ADL	activities of daily living
TOT	task-oriented training
WHO	World Health Organization
WSO	World Stroke Organization
ESO	European Stroke Organization
US VA/DoD	the United States Department of Veterans Affairs and Department of Defense
